# Correcting Hybrid Density Functionals to Model Y6 and Other Nonfullerene Acceptors

**DOI:** 10.1021/acs.jctc.6c00424

**Published:** 2026-08-03

**Authors:** Tom Ward, Isabel Creed, Tim Rein, Jarvist Moore Frost

**Affiliations:** † Department of Chemistry, 4615 Imperial College London, Exhibition Road, London SW7 2AZ, U.K.; ‡ Department of Physics, Imperial College London, Exhibition Road, London SW7 2AZ, U.K.

## Abstract

Recently developed fused-ring electron acceptors such as Y6 (BTP-4F) have strong oscillator strength, good charge-carrier transport, and a small band gap. They therefore have enormous current technical applications to organic optoelectronics, such as solar cells. To design new materials, it would be useful to predict the electronic structure accurately. Because of the large number of atoms involved in representative aggregates of these materials, we need an efficient electronic structure method. Standard density functional theory poorly describes charge-transfer states and was typically parametrized for vacuum calculations of individual molecules. In this work, we tune a range-separated hybrid functional for Y6 and characterize representative dimers extracted from the solid state. We demonstrate that the extensive solvatochromic effects of Y6 are due, in part, to oscillator strength borrowing between the charge transfer and Frenkel excitons. We provide an explanation for the short, optimally tuned range-separation parameter, based on the Penn model for the frequency-dependent dielectric of a semiconductor. We caution that nontuned range-separated hybrids are less accurate than global hybrids for these, and similar, materials. We show how reducing the range-separation length improves the accuracy of standard range-separation functionals without an involved tuning process.

## Graphical abstract

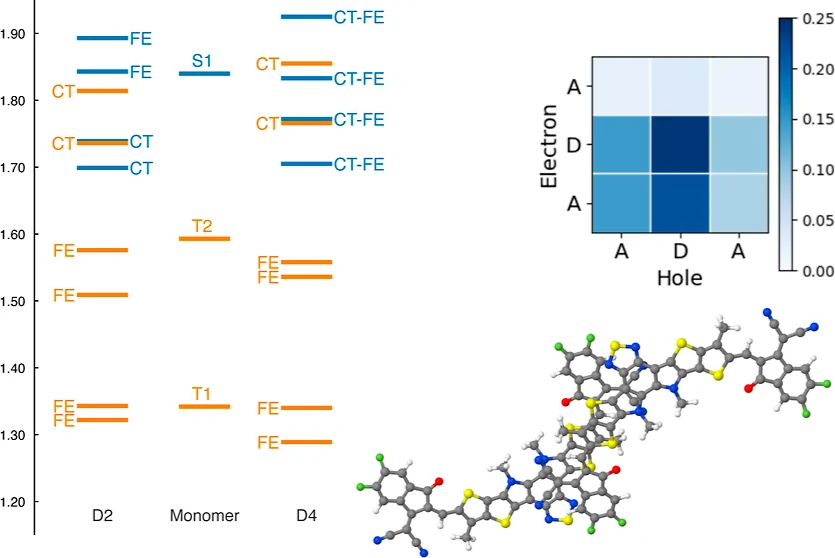

## 1 Introduction

The development of nonfullerene (electron) acceptors (NFAs) has enabled a significant step forward in organic photovoltaic (OPV) power-conversion efficiency. Modern fused-ring electron acceptors (FREAs) have strong absorption, tuned to the near-infrared part of the solar spectrum using donor–acceptor (push–pull) molecular design.

This strong oscillator strength and small band gap, packed into a small volume, directly lead to a large dielectric constant, which has key ramifications for the correct quantum-chemical methods.

The archetypical modern FREA is Y6 (BTP-4F).^1^ Y6 has a banana-shaped but flat A-DA’D-A structure, with the alkyl side chains, required for solubility, kept apart from the solid-state π–π stacking of the fused cores required for high intermolecular mobility. The central unit is electron-deficient benzothiadiazole (A′) fused with electron-rich thieno­[3,2-b]­thiophene-pyrrole (D), linked via conjugated vinyl bonds to electron-poor fluorinated and cyano-substituted indanone (A).^2^

Y6 exhibits good processing behavior with a wide variety of codeposited materials, has strong optical absorption of its own, and seems to possess good charge-carrier transport properties.^2^ Overall, Y6 has become the first-choice electron acceptor in a bulk heterojunction organic photovoltaic.

Neat Y6 films demonstrate unusual photophysics. The solvatochromic shift of Y6 in the solid state is giant. A computational study^3^ predicts that this giant shift is only seen in the singlet state energies, whereas the modest shift of the triplet state energies is consistent with other FREAs. There is some evidence that free charges may be directly generated without an applied bias;^4^ certainly the energetic cost of exciton dissociation into free charges is small.^5,6^ The separation of tightly bound Frenkel-like excitons in organic semiconductors into free charges is the central theoretical mystery of the operation of organic photovoltaics: a naïve model considering a bulk dielectric constant would suggest an exciton binding energy of over an electron volt.

As a simple bilayer with rubrene, Y6 upconverts infrared light into visible (via triplet–triplet annihilation; two photons in for one photon out) with the highest efficiency for a solid-state device.^7^

Often the concept of intermolecular charge transfer (CT) states^4,7–9^ is invoked to explain the unusual photophysics of Y6. As these are intermolecular properties, a theoretical treatment first requires a structural model. Initially, this required molecular dynamic simulations,^10^ but more recent studies^11^ often use the now available high-quality crystal structures^12^ (CSD code OHUBUR).

Charge-transfer states are challenging to describe well with density functional theory (DFT). The self-interaction error in DFT is often corrected by including a fraction of exact exchange from Hartree–Fock. But as these “global” hybrids have the incorrect long-range behavior, CT states can become qualitatively incorrect.

Optimally tuned screened range-separated hybrid (OT-SRSH) functionals were developed explicitly to have a well-balanced description of both local and long-range excitations.^13–16^ They achieve this by having the fraction of Hartree–Fock exchange follow an error function, transitioning from the locally screened (α fraction of Hartree–Fock) domain to a long-range (screened by only the macroscopic dielectric constant, an α + β Hartree–Fock fraction). For vacuum range-separated hybrid (RSH) functionals (assuming ϵ_∞_ = 1 and constrained by α + β = 1), this recovers the full Hartree–Fock limit at long range, whereas for screened range-separated hybrid (SRSH) functionals (ϵ_∞_ > 1), the long-range fraction is reduced by the macroscopic dielectric screening.

Here, we tune the LC-ωhPBE functional. This was previously indicated as having high accuracy for Y5 and ITIC monomers by Franco et al.,^17^ used by Giannini et al.^11^ to model Y6 dimers, and more recently applied to excitons in solid-state Y6 by Akram et al.^18^ This functional has been used to model organic heterojunctions such as the C_60_/pentacene and P3HT/PCBM dimer.^16^ This functional is believed to predict the correct ordering of the CT and FE states in molecular systems.^14–16^

We analyze the excited states predicted by our tuned LC-ωhPBE functional and compare them to untuned, range-separated, and global hybrid functionals. We find that the optimal fits produce a negative beta parameter, leading to a functional where Hartree–Fock is more screened at a longer range. This we link back via a GW picture to the high charge-carrier mobility of the material. Using the simple Penn^19^ model for the dielectric function of a semiconductor, we can predict the range separation parameter directly from the small bandgap.

Our key warning is that “off-the-shelf” range-separated hybrid functionals (such as CAM-B3LYP), which have been developed for typical organic molecules, are actually worse at predicting excited states in modern low-bandgap high-absorbance organic semiconductors than the global hybrids (such as B3LYP). We find that by modifying the range-separation parameter of the popular CAM-B3LYP functional to reflect the small band gap and large dielectric constant of Y6, we are able to reproduce the same correct excited-state properties, ranking, and absolute energy as predicted by our much more involved optimal tuning of LC-ωhPBE. Our key recommendation is that when modeling low-bandgap, large-oscillator-strength, high-dielectric materials (i.e., all modern organic semiconductors), a suitably reduced range-separation parameter is used.

## 2 Methods and Computational Details

### 2.1A Optimally Tuned Range-Separated Hybrid

Optimally tuned (OT) range-separated hybrid functionals empirically impose an Ewald partition of the Coulomb energy operator:^16,20^

1 $$\frac{1}{r}=\frac{\alpha +\beta \times \mathrm{erf}\left(\omega r\right)}{r}+\frac{1-\left(\alpha +\beta \times \mathrm{erf}\left(\omega r\right)\right)}{r}$$

The α, β, and ω parameters are then adjustable for the system of interest.^21^ The parameter ω determines the distance at which one transfers from short-range exchange fraction (α) to long-range exchange fraction (α + β).

For a given value of ω, the exchange correlation energy is an admixture as^21^

2 $$\begin{array}{ll} E_{XC}^{SRSH}= & E_{PBE\left(C\right)}\\ & +\alpha E_{HF\left(X\right)}^{SR}+\left(1-\alpha \right)E_{PBE\left(X\right)}^{SR}\\ & +\left(\alpha +\beta \right)E_{HF\left(X\right)}^{LR}+\left(1-\left(\alpha +\beta \right)\right)E_{PBE\left(X\right)}^{LR}. \end{array}$$

The terms in the summation of eq 2 are (global) GGA/PBE correlation energy, short-range (“SR”) HF and GGA/PBE exchange, and long-range (“LR”) HF exchange and GGA/PBE exchange.

Tuning is achieved by enforcing a generalization of Koopmans’ theorem, where the energy of the Kohn–Sham frontier orbitals reproduces the ionization potential (IP) and electron affinity (EA) of the system, as calculated consistently with a delta-SCF method and the same functional. As proposed by Zheng et al.,^16^ we optimize HOMO and LUMO eigenvalues simultaneously, minimizing a mean squared loss:

3 $$J\left(\omega \right)={\left[\epsilon_{HOMO}\left(\omega \right)+IP\left(\omega \right)\right]}^{2}+{\left[\epsilon_{LUMO}\left(\omega \right)+EA\left(\omega \right)\right]}^{2}$$

We use LC-ωhPBE, a screened range-separated hybrid (SRSH) functional, and so constrain α + β ≡ 1/ϵ_*r*_ ^16,20^ when minimizing *J*(α, β), where ϵ_*r*_ is the bulk optical dielectric constant of the system, having first fitted ω in a vacuum. This allows us to impose the macroscopic behavior of the solid-state material without requiring it to be perfectly recovered from the functional and without requiring a noisy fitting of ω in a polarizable medium. This is in contrast to tuning for RSH functionals, where the constraint α + β ≡ 1 is applied after having fitted ω in a polarizable medium.

Measured values of ϵ_*r*_ for Y6 films range from ϵ_*r*_ ≃ 2.5 ^22^ to ϵ_*r*_ ≃ 6 ^23^ to ϵ_*r*_ ≃ 11.^24^ Following discussion with experimental colleagues, we chose what we felt was a high but still well-founded value of ϵ_*r*_ ≃ 6. We considered the effect of modifying ϵ_*r*_ = 3 – 10 in the Supporting Information (Figure S2) and found that the ordering and other properties of the low-lying CT and FE singlet and triplet states do not depend strongly on the value of the dielectric constant chosen in our tuning.

### 2.2 Wave Function Analysis

To demonstrate that the OT-SRSH functional accurately describes excited states in Y6, we used TheoDORE^25^ to analyze the character of each active state. TheoDORE calculates state properties by considering the ratio of the diagonal and off-diagonal elements of *D*_ab_^0i^ the ground to i-th excited state transition density matrix (TDM) in the orbital basis {a, b}. The orbitals {*a*, *b*} are localized on the molecular fragments *A* and *B*.^25^

For the Y6 dimers, a natural motif for the fragments is the two monomers. One can further naturally subdivide Y6 into two acceptor end units (A) and a donor core (A’DA’).

Two key properties we want to predict are the charge-transfer (CT) character and the extent of localization.

In TheoDORE,^25,26^ the CT character is given by the parameter ω_*CT*_, which corresponds to the proportion of the excitation which occurs between fragments—the degree to which the excitation leads to charge transfer. A value of ω_*CT*_ = 1, therefore, corresponds to a ‘pure’ CT state, while ω_*CT*_ = 0 is a “pure” Frenkel-Exciton (CT) state. Due to CT-FE state mixing at short separations, most excited states of the dimers are not pure FE or CT states. For Y6, this has a considerable effect on the energetic ordering of the singlet and triplet states. Following Zhang et al.,^16^ we consider CT states as those states with ω_*CT*_ ≳ 0.75 and FE states as those states with ω_*CT*_ ≲ 0.25 with all other states being considered “mixed” states.

We define the excitation localization by the participation ratio, ω_*PR*_.^25^ For two Y6 monomers, ω_*PR*_ = 2 corresponds to an excited-state wave function that is entirely delocalized (evenly distributed) across both monomers, while ω_*PR*_ = 1 describes full localization on one monomer.

### 2.3 Computational Details

Gaussian16 ^27^ was used for all of the electronic structure calculations in this work. We use a modest 6-31G­(d,p) basis set. This basis set was chosen after checking basis-set dependence for excited-state energies (shown in the Supporting Information in Figure S1) and tests indicating that calculations converged reliably and smoothly. We use the Tamm-Dancoff Approximation (TDA) in linear-response time-dependent theory for all excited state calculations. This approximation ensures that the singlet and triplet states are ordered consistently.

We studied the Y6 monomer and a set of six contact pair dimers; these dimers were extracted from a solved crystal structure,^28^ with a distance cutoff. Among the unique pairs, eight have a minimum separation of less than 5 Å, defined by Giannini et al.^11^ as “contact pair dimers”, of which we study D1–D6, which have a minimum intermolecular distance of less than 3.5 Å. At these small separations, intermolecular electron transfer integrals become significant and affect the excited state energies.^29^

## 3 Results and Discussion

### 3.1 OT-SRSH for Y6 Dimers

The tuned parameters for representative Y6 dimers and the monomer, alongside the defaults, are given in Table 1. The parameters for different dimers are within 10% of each other, and the monomer-tuned parameters are similarly close. This suggests that the tuned parameters are robust to different dimer configurations. As we only tune on contact dimers in a PCM, we cannot make a strong statement about whether these parameters would also be correct for long-range pairs.

**Table 1:** LC-ωhPBE Default Parameters and Then Our Tuned LC-ωhPBE Functional Parameters for the Y6 Monomer and a Sample of Y6 dimers^11^ ^,^ ^a^

dimer	type	ω (*a*_0_^–11^)	α	β
default	N/A	0.40	0	1
monomer	-	0.140	0.260	–0.093
D1	contact	0.120	0.230	–0.063
D2	contact	0.110	0.250	–0.083
D3	contact	0.110	0.240	–0.073
D4	contact	0.120	0.250	–0.083
D5	contact	0.120	0.240	–0.073
D6	contact	0.120	0.240	–0.073
D7	contact	0.130	0.250	–0.083
D8	contact	0.130	0.250	–0.083
D9	non-contact	0.130	0.250	–0.083
D10	non-contact	0.130	0.250	–0.083

a The units of ω are inverse Bohr radius, *a*_0_^–1^, whereas α and β are unitless fractions. The 6-31G­(d,p) basis set was used throughout. Tuning of α and β was completed in a PCM (using the SCRF method) with ϵ_*r*_ = 6. Note that our predicted β fraction is slightly negative; this means that there is less Hartree–Fock exchange at long range than at short range. The ensemble average of ω for the dimers is 0.122, consistent with a recently published value for Y6 aggregates of 0.127.^18^

A key observation is that the β parameter is negative: screening is greater at a longer range. Though unusual for molecular systems, this is entirely consistent with the formalism and has been observed before.^30^ Here, we connect this to the point of view that the high charge carrier mobility in Y6 provides additional macroscopic screening, like a traditional semiconductor.

We benchmark the excited-state energies from our tuned functional and standard methods against GW/BSE/MM calculations.^11^

We present the mean-squared error for the first six singlet excited states in Table 2. Error is minimized for our tuned SRSH (LC-ωhPBE); it is very notable that nontuned range-separated hybrids demonstrate the largest error, significantly larger than the global hybrids.

**Table 2:** Mean-Squared Error (versus GW/BSE/MM References) Δ_*S*_^TDDFT^ (equation S1) for the First Six Excited-State Singlets in eV

functional	D1	D2	D3	D4	D5	D6
LC-ωhPBE	0.04	0.06	0.05	0.09	0.09	0.12
B3LYP	0.05	0.10	0.09	0.11	0.09	0.12
PBE0	0.10	0.08	0.08	0.11	0.14	0.16
CAM-B3LYP	0.64	0.56	0.56	0.69	0.72	0.75
wB97XD	0.76	0.67	0.67	0.82	0.87	0.92

In the Supporting Information (Figures S3–S5), we show how the energy (E) and extent of charge transfer (ω_*CT*_) of the six lowest singlet and triplet states of the D1, D2, and D4 dimers depend on the functional.

The functionals can be classified on the basis of the ordering of the FE, CT, and mixed CT-FE states. The OT-SRSH, B3LYP, and PBE0 functionals produce a state ordering consistent with the GW/BSE results (private communication with authors of ref 11: they observe no state crossing of the dimer excited states from the PBE0 starting point to the eigenvalue self-consistent GW/BSE/MM results). The default parameter range-separated functionals CAM-B3LYP and wB97XD give an erroneous ordering.

B3LYP is known to underestimate the energy of CT states, particularly in the dissociation of a dimer.^31^ The OT-SRSH functional is expected^14–16^ to correctly order CT and FE states. Therefore, agreement of our OT-SRSH to B3LYP initially gave us concern. In the Supporting Information (Figures S7 and S8), we show that the OT-SRSH functional is able to obtain the correct behavior of the ordering of the CT and FE states during dimer dissociation, whereas B3LYP does not. It is a happy coincidence that in these dimers the global hybrids are correctly balanced.

For our tuned SRSH to be used in the study of Y6 solid and thin films, the tuning parameters must be transferable, i.e., the same parameters should work consistently well on monomers, dimers, and larger aggregates. This is to avoid the need for repeating the tuning process on different structures and to avoid having to extrapolate from increasingly large aggregates to the thermodynamic limit. To demonstrate transferability of the tuned functional, we compare the singlet and triplet excited-state properties for all contact pair dimers when the calculations are run using the tuned parameters for the dimer being considered and when the calculations are run using the monomer tuning. We found that the state properties in both cases are practically identical. In the Supporting Information (Figure S6), we show detailed analysis of this for the D2 dimer, with similar results being found for all dimers examined.

A small reorganization energy enables large charge mobility^32,33^ and reduces nonradiative losses in OPVs.^34^ Given the importance of the reorganization energy, we validate the use of our OT-SRSH in calculating the reorganization energy of the monomer using both Nelsen’s four-point method^35^ and Reimers’ normal-mode-resolved approach.^36^ These data are shown in Figures S10 and S11 of the Supporting Information. We find that our tuned SRSH has similar values of reorganization energy to the commonly used functionals PBE0 and B3LYP; our tuning for excited states has not made the prediction of the potential energy surfaces erroneous. All tested functionals are capable of capturing the relatively small reorganization energy of the Y6 first-singlet, which is notably smaller than for the cation or anion.

### 3.2 Excited-State Properties of Y6

Having somewhat validated our OT-SRSH functional, we now move on to considering the properties of the excited states of the contact dimers. Our full analysis of the excited-state properties of the dimers is in the Supporting Information (Figures S13, S16, and S21).

Dimers D2 and D4 represent two distinct dimer behaviors in the Y6 crystal. We show their Jablonski diagram alongside the monomer in Figure 1.

**Figure 1:**
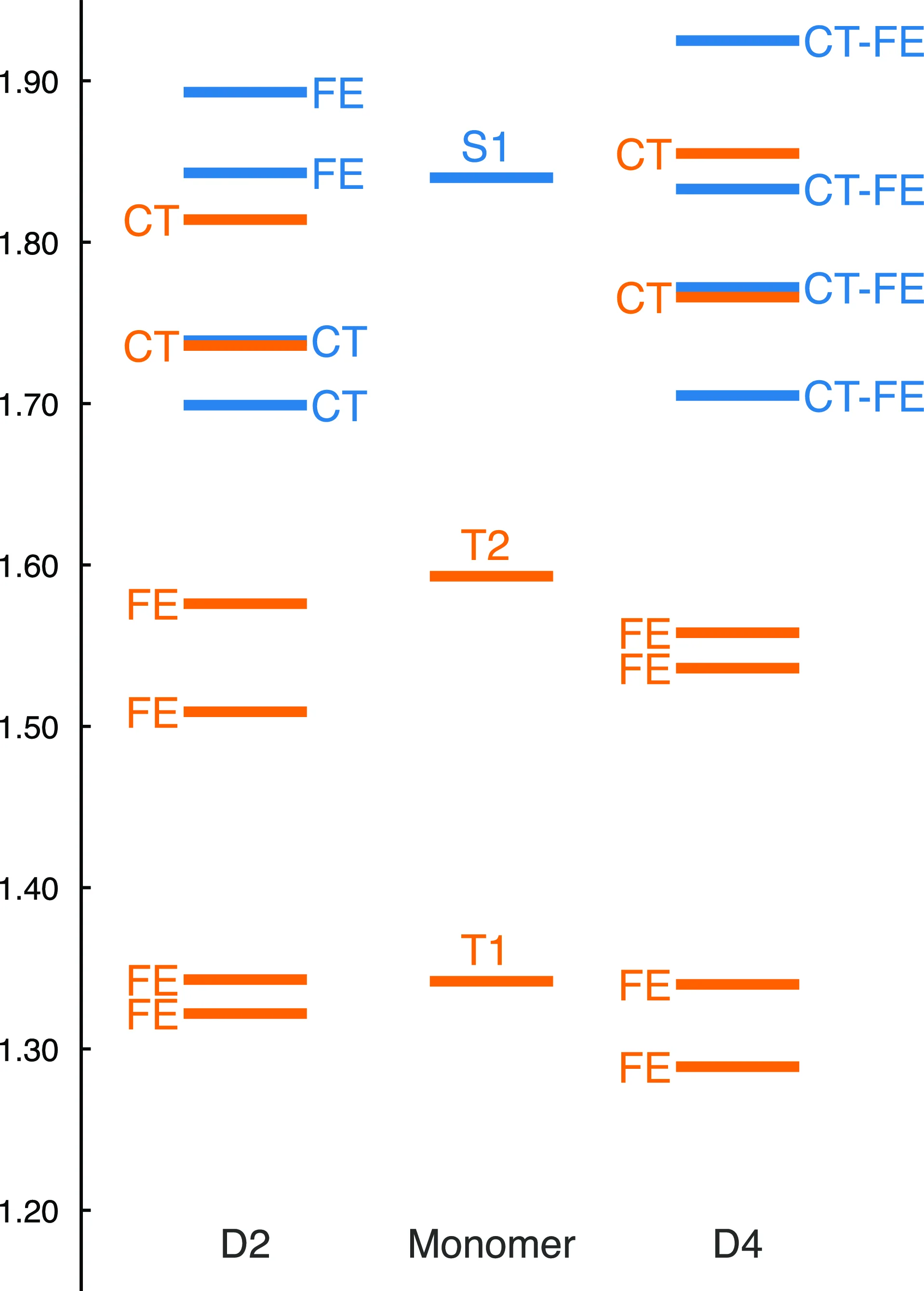
Jablonski diagram of the D2 dimer (example of the J aggregate), monomer reference, and D4 dimer (example of the H aggregate). Triplet states are in orange, and the singlet states are in blue; the label reflects the CT character; energy is in eV.

In the D2 dimer, the two lowest-in-energy singlet states are CT states, followed by two higher-in-energy FE states, while for the D4 dimer, all four lowest-energy singlet states are mixed CT-FE states. The strong dependence of the FE-CT mixing in the singlet states on the class of dimer is shown explicitly by examining ω_*CT*_ in the Supporting Information (Figures S12 and S13). By contrast, the four lowest-in-energy triplet states are FE states, followed by two higher-in-energy CT states. In the Supporting Information, we show (in Figures S12 and S13) that the triplet-state FE-CT mixing has a much smaller dependence on the type of dimer than the singlet states. We find that the extent of delocalization of the triplet excited states, as probed by ω_*PR*_, is ω_*PR*_ ≃ 2 in the D2 dimer to ω_*PR*_ ≃ 1 in the D4 dimer, whereas singlet states are consistently delocalized (ω_*PR*_ ∼ 2).

The triplet CT states of both dimers lie at higher energies than the singlet CT state/mixed CT-FE states. This is because the singlet CT states are stabilized by this mixing. In contrast, triplet CT states are destabilized to higher energy states. Similar behavior is observed in interfacial dimers of Y6/ZR1,^37^ where it was proposed that significant CT-FE mixing causes singlet–triplet inversion. This inversion of singlet/triplet interfacial CT states has been hypothesized to reduce recombination in Y6 OPVs.

The key role of the CT-FE mixing in the bathochromatic shift of the singlet states has been noted.^11^ However, in this study, it is predicted that the CT states of the dimer lie above the FE states. This means that they failed to predict the existence of the polaronic states of charge located on neighboring Y6 molecules, believed to be present in their experimental data.^11^

Our tuned OT-SRSH predicts that these spectroscopic features are charge-transfer states formed by contact dimers. There is some evidence for such low-lying CT states being observed in electroabsorption (EA) measurements.^38^

### 3.3 Fixing Range-Separated Hybrids without Tuning

Our tuned parameters are consistent between the monomer and dimers. The key change is the larger dielectric constant screening the Coulomb interaction (thus reducing Hartree–Fock exchange) and a shortened range-separation parameter.

GW theory calculates the full dielectric function (frequency-dependent) in the random phase approximation (RPA) and then uses this to screen the Coulomb (Hartree–Fock) interaction.

Hybrid density functionals can be considered a static approximation to this, where the fraction of exact exchange (an empirical parameter) then presents as being 1/ϵ_∞_. From this perspective, PBE0 (25% exact exchange) can be interpreted as implicitly assuming an optical dielectric constant of 4. The interpretation for functionals with more complex admixtures of exchange–correlation (such as B3LYP) is not so clear.

Screened range-separated hybrid density functionals can be considered an extension possessing a model of the inverse dielectric function: that of the error function. At very short distances (high frequencies), the dielectric response of the material is not fast enough to screen the Coulomb interaction; at large distances, this tends to the macroscopic 1/ϵ_∞_.

As Y6 has such strong (high oscillator strength) and low energy excitations, the dielectric constant is far larger than typical for organic matter (≈2) and other organic electronic materials (≈3). By the Thomas–Reiche–Kuhn f-sum rule, this is a repercussion of the total oscillator strength (a conserved quantity) being shifted down in energy, and so the screening (linked to the fastest response of the material) distance becomes shorter.

In 1962, Penn^19^ provided a simple but fruitful model for the dielectric function of a semiconductor. This model includes the key physics of the presence of the fundamental gap and parabolic bands and permits Umklapp scattering processes. A key result is that it predicts ϵ_*∞*_ from the gap *E*_*g*_,

4 $$\epsilon_{\infty }\approx 1+{\left(\frac{\hbar \omega_{p}}{E_{g}}\right)}^{2}$$

where ω_*p*_ is an effective plasma frequency constrained by the Thomas–Reiche–Kuhn *f*-sum rule (oscillator-strength sum)^39^ and *E*_*g*_ is an effective interband gap.

Hence, small *E*_*g*_ and/or large low-energy oscillator-strength density leads to a large ϵ_∞_, as observed in Y6.

Penn provides an interpolated dielectric function later reinterpreted in a simple Lorentzian form,^40^

5 $$\epsilon \left(q\right)=1+\frac{\epsilon_{\infty }-1}{1+q^{2}/q_{s}^{2}}$$

with ϵ_∞_ as in the previous equation and *q*_*s*_ being an effective screening wavevector, $q_{s}^{2}=2\mu E_{g}/\hbar^{2}$ set to where the electron kinetic energy starts to excite across the effective interband gap. Clearly, we go from full ϵ_∞_ screening at *q* = 0 to unscreened at large *q*.

The half-maximum midpoint, i.e., the characteristic screening distance, of this Lorentzian is at *q* = *q*_*s*_.

Now going back to the SRSH form for the range separation, the Fourier transform of erf­(ω*r*)/*r* yields a Gaussian reciprocal-space exchange fraction 1 – exp­[−*q* ^2^/(4ω^2^)].

The midpoint of this screening function is at $q=2\omega \sqrt{\ln2}\approx 1.67\omega$ .

Matching this to the Penn half-maximum at *q*_*s*_ gives a direct estimate for the range-separation parameter as

6 $$\omega \approx \frac{q_{s}}{2\sqrt{\ln2}}\approx 0.6q_{s}$$

In atomic units (*ℏ* = 1, distance as Bohr, energies as Hartree), we can therefore predict our range-separated parameter directly from the Penn model as

7 $$\omega \left[a_{0}^{-1}\right]\approx \frac{q_{s}}{2\sqrt{\ln2}}\approx 0.6q_{s}=0.6\sqrt{2\mu \left[m_{e}\right]E_{g}\left[Ha\right]}$$

The effective mass arises from the random-phase approximation integrating over a parabolic band in the Penn model. For organic semiconductors, this approximation is crude. We could probably improve this approximation with an explicit calculation of the electronic structure, but we leave this for future work.

For low-bandgap nonfullerene acceptors such as Y6, assuming an effective mass of 0.3 *m*_*e*_, with a bandgap of 1.3 eV (0.05 Ha), this gives $\omega =0.10a_{0}^{-1}$ , in striking agreement to the value from our tuning approach.

Perhaps more important than this quantitative prediction is the scaling $\omega \propto \sqrt{\mu E_{g}}$ . Assuming broadly similar effective mass within an organic semiconductor family, the dominant variation is through $\sqrt{E_{g}}$ . This directly explains why small-bandgap materials (i.e., almost all modern organic semiconductors) require a smaller range-separation parameter than the defaults tuned for typical organic molecules.

With this information, we now try and modify a popular range-separated hybrid, CAM-B3LYP. We retain the default exchange fractions (α = 0.19, α + β = 0.65) and change only the range-separation parameter from ω = 0.33 to 0.10*a*_0_^–1^ (we denote this as ‘CAM-B3LYP-tuned’ in our figures). Figure 2 shows that the resulting excited-state properties are in excellent agreement with the full optimally tuned SRSH functional, despite the substantial differences in the long-range exchange fraction (0.65 vs 1/ϵ_∞_ ≈ 0.17). This indicates that the key dimer photophysics requires just a reduction in the range-separation parameter, rather than being directly affected by the dielectric constant of the material. For larger assemblies, it is likely that the long-range screening (macroscopic dielectric constant) becomes more important, and so the functional should have a tuned α + β parameter.

**Figure 2:**
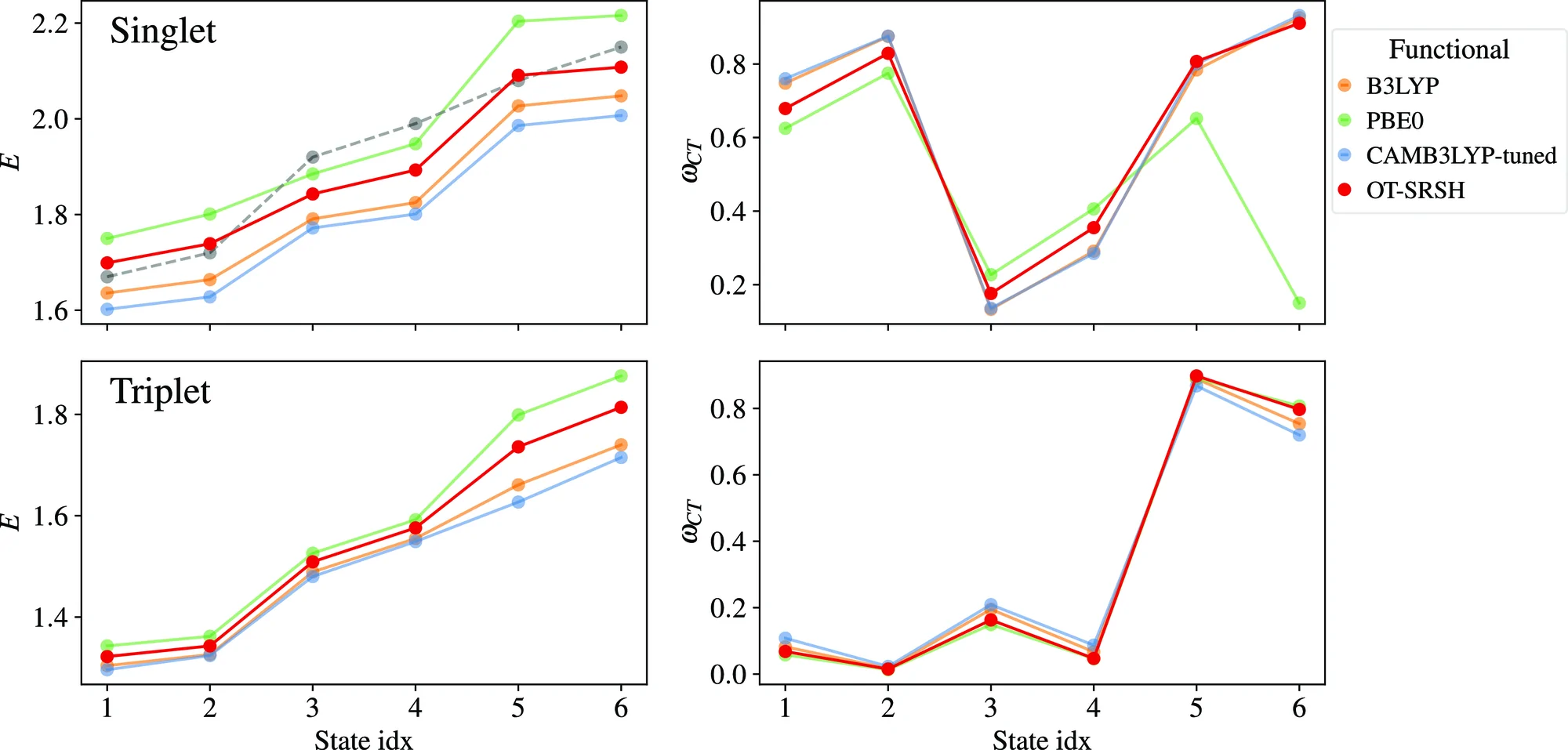
Comparison of the energies (*E*) and extent of charge transfer (ω_*CT*_) given by OT-SRSH, B3LYP, PBE0, and CAM-B3LYP-tuned (CAM-B3LYP with a changed range-separated parameter).

## 4 Conclusion

We tuned a range-separated hybrid functional to model Y6 in the solid state and used this to model the excited states of the contact-pair dimers from the recent high-quality crystal structure.^11,12^

Kasha’s model of H (cofacial), J (head-to-tail), and V (oblique) aggregates classifies the behavior of the contact pair dimers well, with D1, D2, and D4 exemplifying the class. The degree of delocalization and FE-CT mixing varies considerable between the classes.

In the triplet manifold, for all six studied dimers, the first four excited states are Frenkel excitons (FEs), followed by two charge-transfer (CT) states. The triplet states have modest CT-FE mixing and have varying degrees of localization: the D2 dimer states are fully delocalized; the D4 dimer states are localized to a single monomer. By contrast, the character of the singlet excited states is highly dimer-dependent. For D1 and D2, the first two states are charge-transfer in character, followed by two FE states. D4 demonstrates mixed CT-FE states.

This extent of CT-FE mixing is a strong function of the dimer geometry, and we believe that this plays a significant role in the strong solid-state shift of the singlet states observed in Y6. The larger solid-state shift in the singlet states is not just due to the additional delocalization but also due to the larger amount of FE-CT mixing in the singlet states.

We predict the inversion of the singlet and triplet CT states in all of the Y6 contact pair dimers examined. In previous work,^37^ the singlet–triplet inversion has been proposed to reduce the triplet loss mechanism in organic photovoltaics.

We demonstrate that off-the-shelf range-separated hybrid functionals have the incorrect parametrization for strongly absorbing low-bandgap organic semiconductors, such as Y6. For Y6, this means that nontuned range-separated functionals are less predictive than global hybrid functionals. Rather than an involved self-consistent fitting procedure, for CAM-B3LYP, we show that a simple reduction of the range-separation parameter fixes most qualitative and quantitative errors.

By returning to the original motivation for range-separated hybrid functionals and comparing this to frequency-dependent screening in GW theory and building on Penn’s^19^ simple semiconductor dielectric model, we provide a direct prediction of this range-separation (screening length) in terms of the bandgap and effective mass of the material under study. This model suggests that modern low-bandgap high-absorption organic semiconductors would be well described by range-separated hybrid functions with a corrected screening length of $\omega \approx 0.10a_{0}^{-1}$ .

Input files and tuning scripts implementing these workflows and analysis are available as a repository on Figshare.^41^

## Supplementary Material



## Notes

Additional tests of convergence, comparison to standard DFT functionals, excited-state property with dimer separation, checks of reorganization, excited-state properties of the different dimers, corresponding figures, and explanatory text (PDF)
