# Supplementary material for: Correcting Hybrid Density Functionals to Model Y6 and Other Nonfullerene Acceptors
Source: J Chem Theory Comput. 2026 Aug 3;22(16):8433–40. doi: 10.1021/acs.jctc.6c00424 (PMC13523631; doi:10.1021/acs.jctc.6c00424)
Supplement: Supplementary file 1 [file ct6c00424_si_001.pdf]

Supplementary Information: Correcting hybrid density functionals to model Y6 and other non-fullerene acceptors

Tom Ward *[1,*] Isabel Creed [1,*] Tim Rein [2,1] and Jarvist Moore Frost [1,2]
[1] Department of Chemistry, Imperial College London, Exhibition Road, London SW7 2AZ, UK
[2] Department of Physics, Imperial College London, Exhibition Road, London SW7 2AZ, UK
(Dated: May 28, 2026)

S1. BASIS SET CONVERGENCE

Before we tuned the OT-SRSH functional, we wanted to examine how the Y6 dimer singlet and triplet states converged with basis set for standard functionals. We found for all the functionals examined (B3LYP, PBE0, CAMB3LYP and wB97XD) the excited state properties were practically converged for 6-31G basis set, and so this was the basis set used to tune our OT-SRSH. This convergence for CAMB3LYP and D2 dimer is shown in figure S1, similar behaviour is seen for other functionals and dimers.

S2. MEAN SQUARED ERROR

We calculate our mean-squared error as,

$$\Delta_S^{TDDFT} = \sqrt{\frac{1}{6}\sum_{n=1}^{6}\left(E_{S_n}^{TDDFT} - E_{S_n}^{GW/BSE/MM}\right)^2}. \tag{S1}$$

S3. EFFECT OF CHANGING DIELECTRIC CONSTANT

The reported experimental value of ϵ_r for Y6 films ranges from $\epsilon_r \simeq 2.5$[S1] to $\epsilon_r \simeq 6$[S2] to $\epsilon_r \simeq 11$[S3].

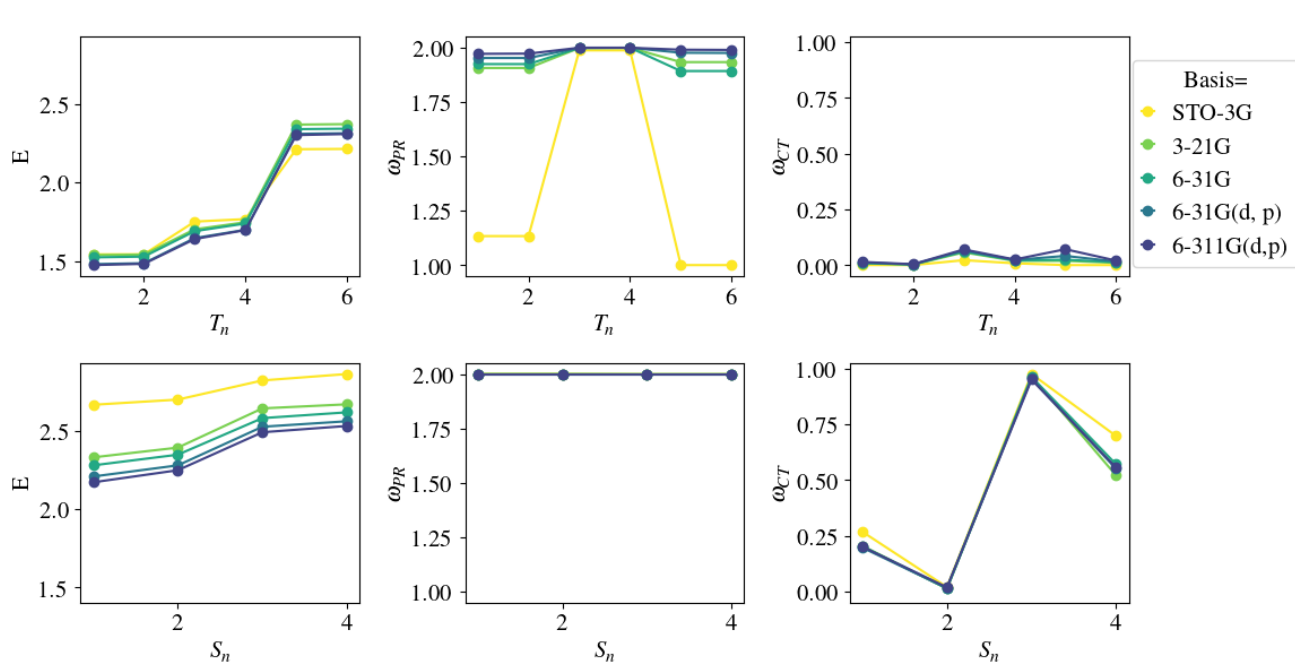

FIG. S1. Figure to show how the triplet (top panels) and singlet (bottom panels) excited states of the D2 dimer using CAMB3LYP functional vary with basis set.

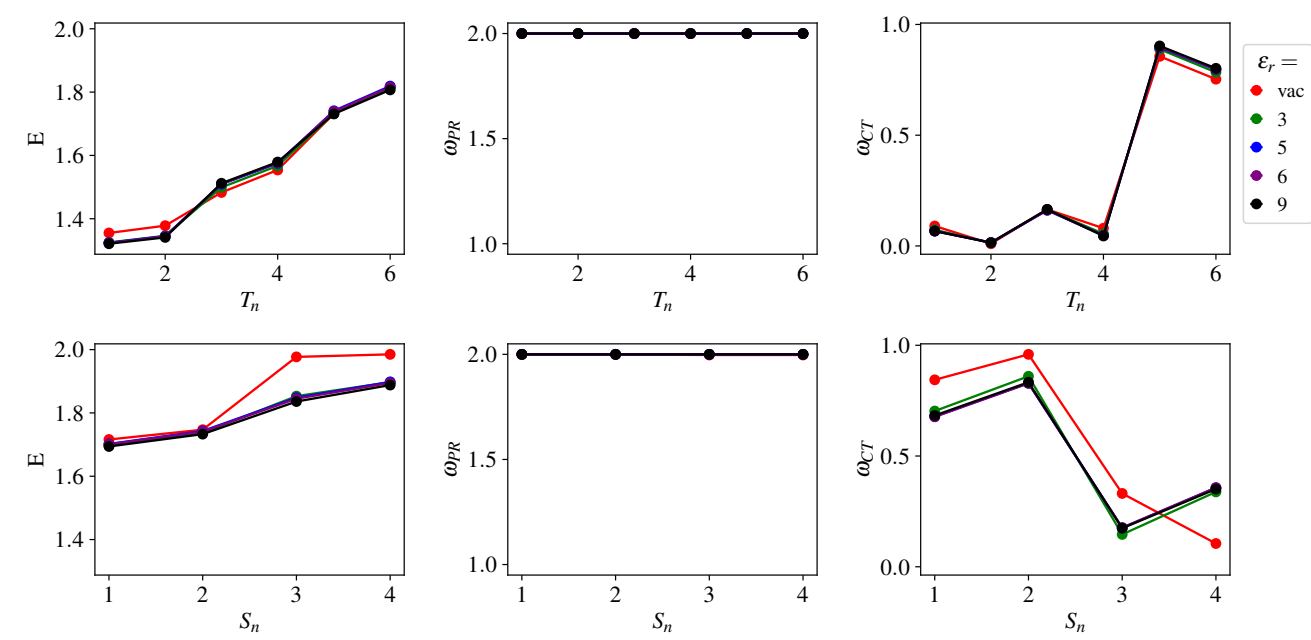

FIG. S2. The properties of the singlet and triplet states of the D2 dimer depend on the dielectric constant ϵ_r used in the tuning of the OT-SRSH and in the PCM.

Given the uncertainty in the literature for the values for the dielectric constant of Y6, we have examined the effect of changing the dielectric constant used in the tuning of our OT-SRSH functional and the corresponding PCM has on the properties of the singlet and triplet states, shown in figure S2.

Figure S2 shows that within sensible range of dielectric constants of Y6, the properties of both the singlet and triplet excited states of the D2 dimer remain practically identical to those shown in the paper.

S4. COMPARISON OF DIFFERENT STANDARD FUNCTIONALS

To validate our tuning, the energies of singlet excited states given by the LC-ωhPBE functional were benchmarked against the results from GW/BSE/MM calculations [S4]. This comparison is shown in figures S3, S4 and S5 for the contact pair dimers D1, D2 and D4. These figures demonstrate good agreement between the tuned functional and the higher level GW/BSE/MM method. We find similarly good agreement results for all the other dimers.

Considering the results shown in figures S3, S4 and S5 we see that the sample of functionals can be broken into two groups based on the energies and CT character of the singlet and triplet excited states in the D1, D2 and D4 dimers.

For our OT-SRSH, together with the global PBE0 and B3LYP functionals, the lowest energy singlet excited states of the D1 and D2 dimer are 2 CT states ($\omega_{CT} \gtrsim 0.75$) followed by 2 FE states ($\omega_{CT} \lesssim 0.25$). In

* These two authors contributed equally

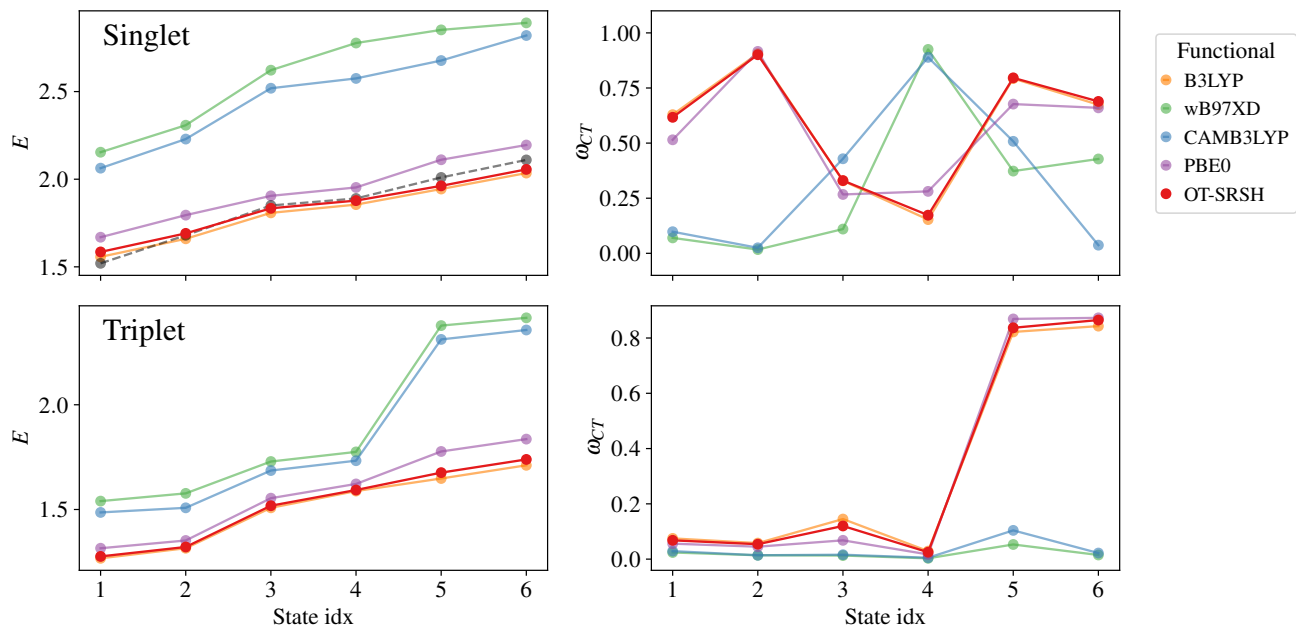

FIG. S3. The energies (E) and the extent of charge transfer (ω_{CT}) for the different singlet and triplet excite states of the D1 dimer changes with functional in PCM with ϵ_r = 6. The black dotted line is the reference GW/BSE/MM calculation[S5].

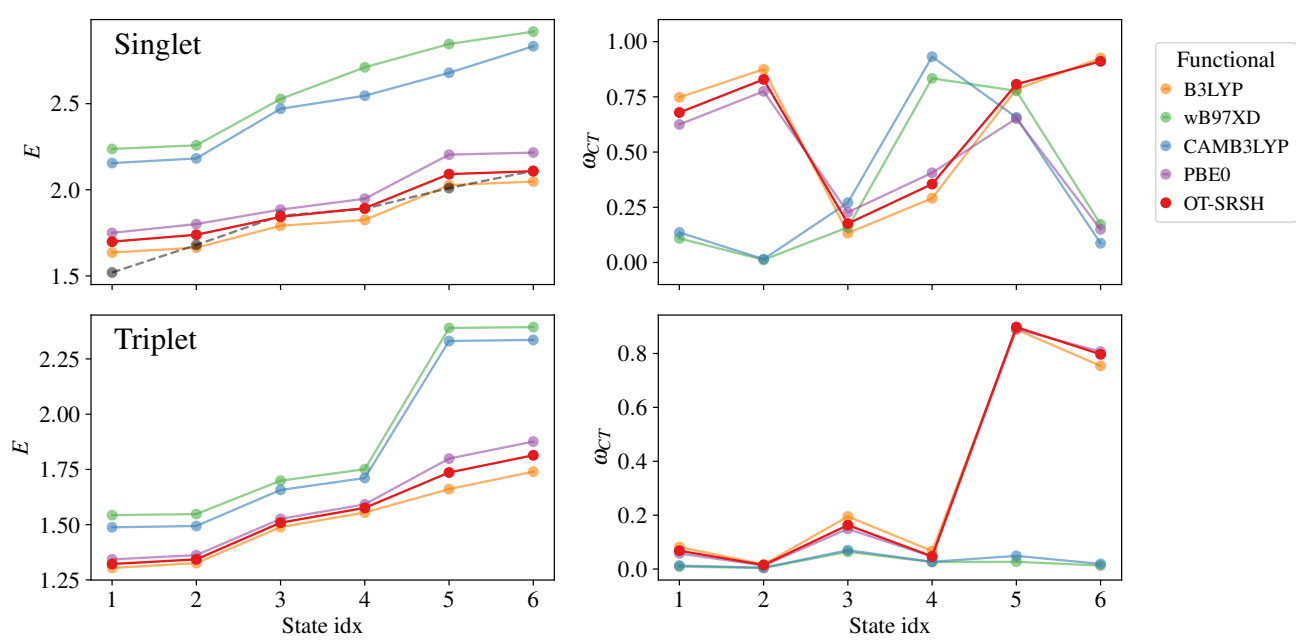

FIG. S4. The energies (E) and the extent of charge transfer (ω_{CT}) for the different singlet and triplet excite states of the D2 dimer changes with functional in PCM with ϵ_r = 6. The black dotted line is the reference GW/BSE/MM calculation[S5].

contrast, for CAM-B3LYP and wB97XD the lowest energy singlet excited states are 2 FE states followed by 2 CT states. By contrast, for the D4 dimer, the OT-SRSH, B3LYP and PBE0 predict mixed CT-FE states with $\omega_{CT} \sim 0.5$ for all the four lowest energy singlet states, whilst for CAM-B3LYP and wB97XD all four lowest energy singlet states are FE states with $\omega_{CT} \lesssim 0.25$.

A similar trend is observed for the triplet excited states of the D1, D2 and D4 dimers, where for OT-SRSH, PBE0, and B3LYP the first 4 lowest excited state are FE states, followed by two higher in energy CT states. For CAM-B3LYP and wB97XD the 6 lowest energy triplet states are all FE states. It is perhaps no surprise that the hybrid functionals CAM-B3LYP and ωB97XD exhibit such different results to the OT-SRSH functional. With range-separation parameters of $\omega = 0.33a_0^{-1}$ and $\omega = 0.20a_0^{-1}$ respectively [S6], which differ greatly to $\omega = 0.11 - 0.13a_0^{-1}$ for the OT-SRSH, significant difference in the electronic structure predicted by these two groups of functionals is expected.

The results of our tuning work and benchmarking demonstrate the functional dependence of both the ordering and properties of the excited states. The CAM-

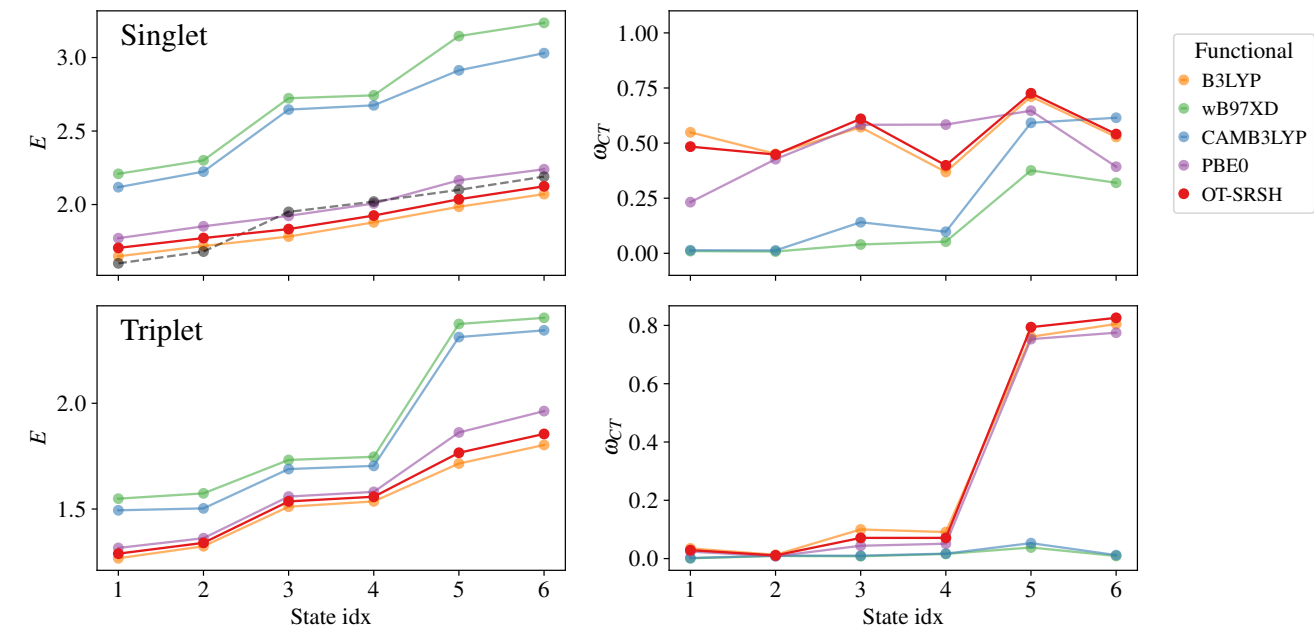

FIG. S5. The energies (E) and the extent of charge transfer (ω_{CT}) for the different singlet and triplet excite states of the D4 dimer changes with functional in PCM with ϵ_r = 6. The black dotted line is the reference GW/BSE/MM calculation[S5]..

B3LYP functional, commonly used in the study of organic systems where CT and FE states are present, disagrees with the results from calculations using the OT-SRSH functional, and the energies given by GW/BSE theory. We therefore advise caution when using the CAM-B3LYP functional in the study of Y6 electronic structure and photophysics.

One noticeable result from this work is the similarity between the results for the OT-SRSH functional and B3LYP. This may be cause for initial concern, since B3LYP is known to underestimate the energies of the CT states[S7–S9], particularly on dissociation of the dimer [S10]. However, we shall see in a later section that our OT-SRSH does not suffer from same issues inherent in the B3LYP functional. These results provide evidence that our optimally tuned SRSH correctly captures the ordering of the CT and FE states, and are robust to different morphologies, whereas it is a happy coincidence that B3LYP captures the correct physics at the dimer separation.

A. Size extensivity of OT-SRSH state

For the OT-SRSH to be used in the study of Y6 solid and thin films, the tuning parameters must be size extensive, i.e. the parameters should provide consistently accurate excited state results when applied to larger aggregates. This is key to avoid the need for repeating the tuning process on various sized molecular aggregates that are used as surrogates for the solid in the ‘thermodynamic limit’.

To demonstrate the size extensivity of the tuned functional, the excited state properties for dimer D2 are compared for when a) calculations are run using the tuned parameters for dimer D2, and b) calculations are run using parameters tuned for the monomer. The results of this comparison (Fig. S6) show that for both singlet and triplet excited states, the state energies and the extent of charge transfer (ω_{CT}) are independent of whether the

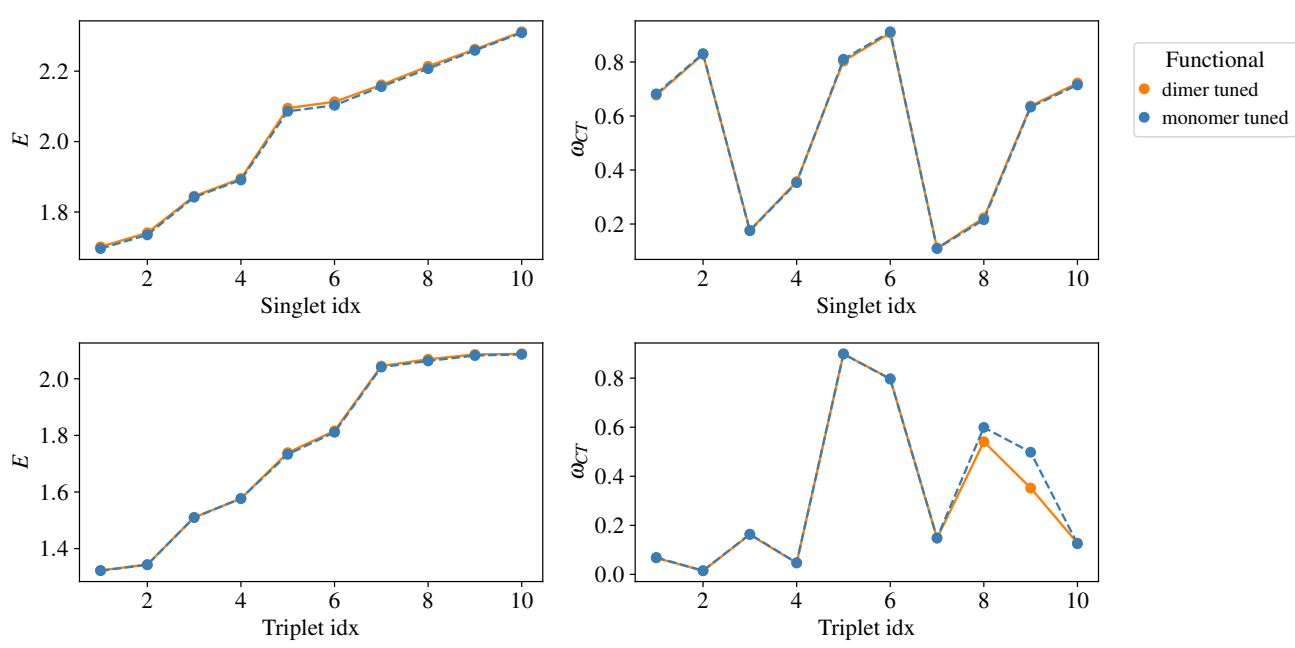

FIG. S6. The singlet and triplet excited state energies (E) and extent of charge transfer (ω_{CT}) of the D2 dimer, where the OT-SRSH parameters used in the calculation were obtained by tuning on the monomer (blue) and the dimer D2 (orange) respectively.

tuning was done on the monomer or dimer. This demonstrates the size extensivity of the tuned parameters. Additionally, we find that for OT-SRSH tuned on both the monomer and the dimer, the singlet and triplet excited states in dimer D2 have $\omega_{PR} \simeq 2$, meaning the excited states are completely delocalised across both monomer units. We find similar results for all the contact-pair dimers. Therefore, we expect that for calculations on Y6 molecular aggregates, the OT-SRSH parameters tuned on the monomer can be applied to larger molecular aggregates.

A similar result using different analysis was found in the recent paper by Akram et al.[S5].

B. Properties of states with increased dimer separation

In addition to size extensivity, it is important that the tuned functional is able to capture the correct ordering of the CT and FE states and the extent of delocalisation/size of the exciton in Y6 crystals. To probe this, we consider how properties of the first 4 singlets and 6 triplets, extent of charge transfer and delocalisation, change as the separation distance between the two dimers increases, both for our OT-SRSH functional (figure S7) and B3LYP (figure S8).

Physically, we would expect the excited states of the dimer to localise in separate monomer units as the dimer distance increases, with $\omega_{PR} \to 1$ at large separations. We expect that this will happen faster for the triplet and CT states than for the FE states, due to Förster term facilitating easier delocalisation in FE states than other excitation[S11].

Furthermore, for both our OT-SRSH and B3LYP, if the correct physics is captured, we would expect that whilst the charge transfer states are the lowest energy singlet states at short separations for the Y6 dimers, as the separation increases, these states would become increasingly higher in energy. Initially, the S_1 and S_2 states would move from being predominantly CT states to predominantly FE states, meaning the S_3 and S_4 states would move from FE states to CT states. As the distance between the monomers increases further, we would expect that all four singlet states would be FE exciton states.

Similarly, for the triplet states for both our OT-SRSH and B3LYP functionals, if the correct physics is captured, at short separations we expect from our previous calculations that first four excited states are all FE states, followed by two higher in energy CT states, and we would expect that as monomer separation increased eventually all triplet states would be FE states.

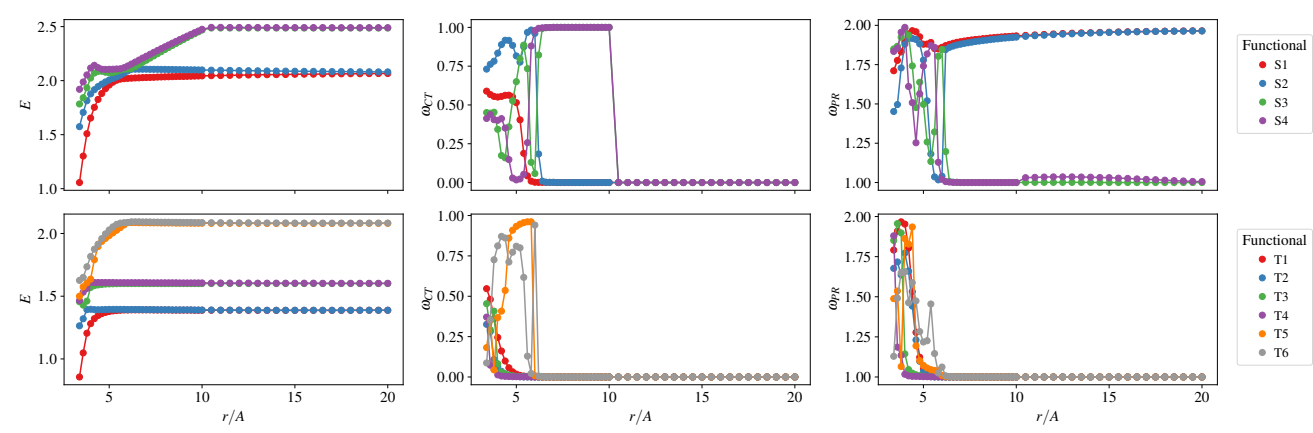

FIG. S7. The energy (E), CT character (ω_{CT}) and extent of delocalisation (ω_{PR}) of the singlet (top panels) and triplet (bottom panels) excited states of the Y6 dimer change with the separation between the monomer units for our OT-SRSH functional.

Fig. S7 shows that the tuned OT-SRSH functional is able to describe the behaviour of the CT character of the singlet and triplet states (ω_{CT}) as the monomer separation is increased. In contrast, figure S8 shows that the B3LYP functional is unable to fully describe this behaviour. In this case, the lowest two singlet states remain CT states even at a monomer separation of 20 Å which is not physical. This leads us to caution the use of B3LYP functional in examining the CT character of larger size aggregates of Y6 or other OPV systems. It is, however, clear that our OT-SRSH could be used to look at CT states in larger aggregates.

We now consider whether any of the functionals considered in this paper are able to describe the physics of FE states at large separations. Figures S7, S8 and S9 show that at large separations for OT-SRSH, B3LYP and

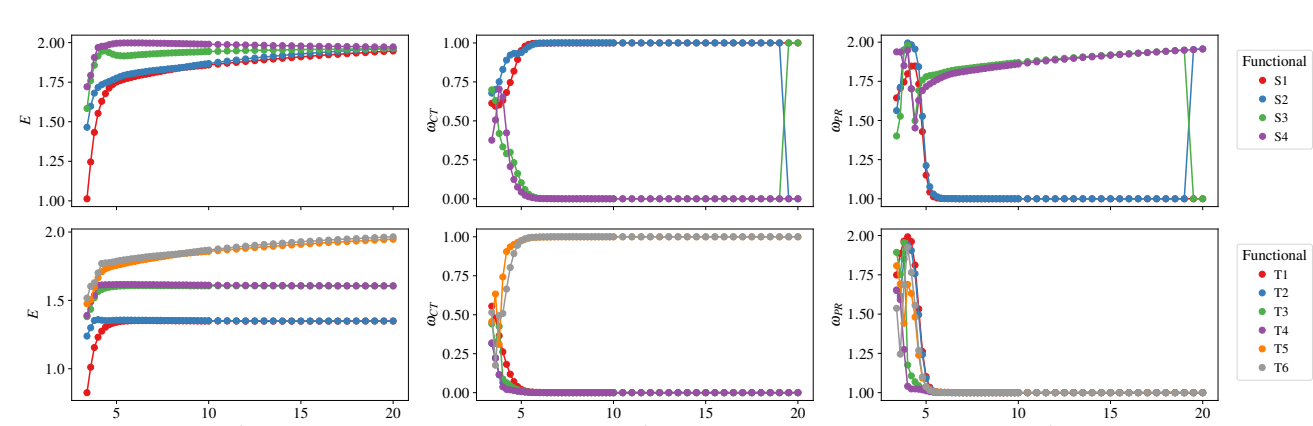

FIG. S8. The energy (E), CT character (ω_{CT}) and extent of delocalisation (ω_{PR}) of the singlet (top panels) and triplet (bottom panels) excited states of the Y6 dimer change with the separation between the monomer units for the B3LYP functional.

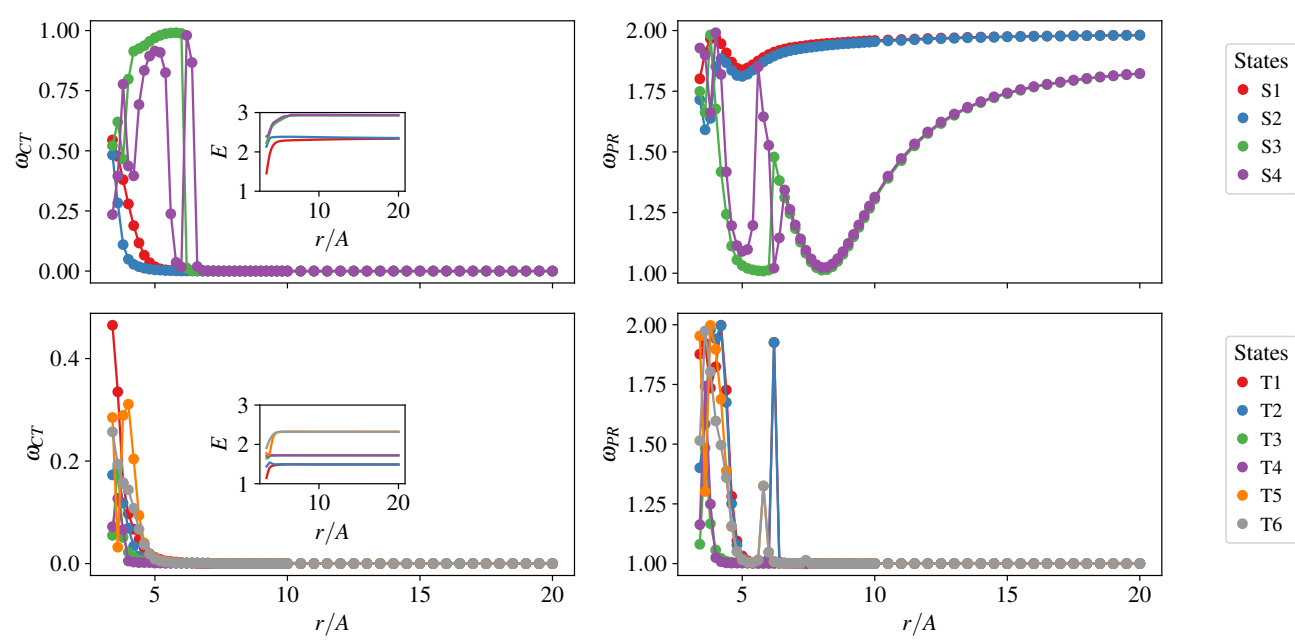

FIG. S9. The energy (E), CT character (ω_{CT}) and extent of delocalisation (ω_{PR}) of the singlet (top panels) and triplet (bottom panels) excited states of the Y6 dimer change with the separation between the monomer units for the CAM-B3LYP functional.

CAM-B3LYP functional, some of the FE singlet states of the D2 dimer remain delocalised even at unphysical separations of 20 Å. Further work is therefore clearly needed to develop a functional capable of capturing both the CT character of the states and delocalisation of the singlet states, and we caution against using the OT-SRSH functional when examining the FE exciton size of Y6 molecular aggregates.

C. Reorganisation energy

When a molecule undergoes an electronic transition, the electron density changes, so that the equilibrium geometry in the new electronic state usually differs from the equilibrium geometry in the initial state, reflecting the change in the electrostatic distribution between electrons and nuclei. The energy associated with the rearrangement of nuclei as a result of an electronic transition is called the reorganisation energy and it plays a crucial role in enhancing efficiencies in organic semiconductors [S12–S14].

Several computational approaches can be used to determine the reorganisation energy, which is conventionally partitioned into an intramolecular component (inner reorganisation energy) and an extramolecular component (outer reorganisation energy). Our focus will be solely on the former. The most common technique for calculating the total intramolecular reorganisation energy is Nelsen's 4-Point (4P) method [S15], which requires four single-point energy calculations performed at the equilibrium geometries of the two electronic states and their respective Franck-Condon geometries, accounting for both forward and backward transitions. However, as the 4P method only yields the total value, an alternative approach developed by J. R. Reimers [S16] is often utilised to resolve the individual vibronic coupling strength contributed by each normal mode to the overall reorganisation energy.

To validate the tuning approach and to ensure that

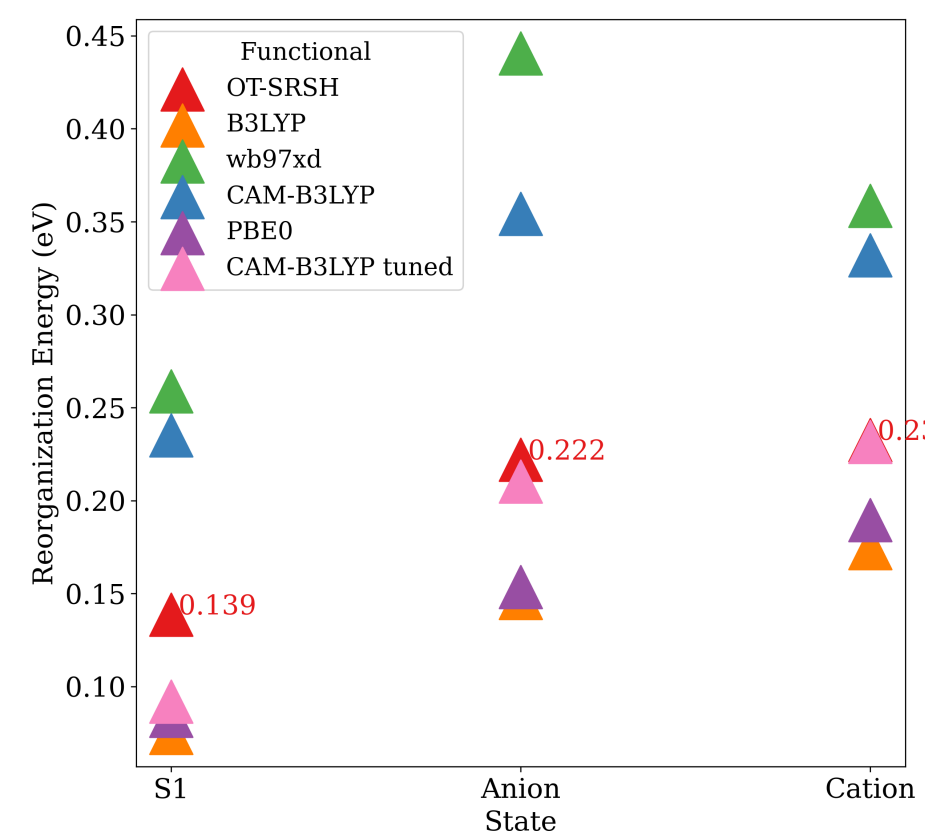

FIG. S10. Reorganisation energies for the first excited state of the different functionals for the Y6 monomer in vacuum, as well as for the anion and cation. Values were obtained via the 4P-approach.

it yields sensible reorganisation energy values, we first calculated the intramolecular S1 excited state reorganisation energy, alongside the hole and electron reorganisation energies (c. fig. S10), for the Y6 monomer in vacuum using the 4P approach. The overall trend confirms that Y6 exhibits a very low excited state reorganisation energy for S1, which notably lies below both its hole and electron reorganisation energies. Furthermore, these results demonstrate for the OT-SRSH reasonable agreement with experimentally determined values, which typically fall around 85 meV [S17] - 103 meV [S18]. Across the excited and charged states, the reorganisation energies predicted by B3LYP, PBE0, and tuned CAM-B3LYP are largely consistent with the OT-SRSH results. In contrast, ωB97X-D and standard CAM-B3LYP yield distinct absolute values, forming a separate cluster. To further test the tuning, it is instructive to examine the normal mode contributions to the reorganisation energy using the method developed by Reimers [S16]. Since the normal modes are fundamentally derived from the diagonalisation of the Hessian matrix, analysing their individual contributions allows us to gain deeper insight into the underlying electronic structure calculated by the different functionals, moving beyond an assessment based solely on total energy values.

Figure S11 presents the calculated normal mode contributions to the reorganisation energy using the DUSHIN code by Reimers' which are normalised to the mode value that couples the strongest. The close agreement between the total reorganisation energies obtained by the 4P approach and by summing the contributions of all normal modes across the different functionals validates the use of the harmonic approximation in these calculations. Furthermore, all functionals consistently identify the primary vibrational contribution occurring near 1600 cm^{-1}, which corresponds to the breathing mode of the $DA'D$ structure. Interestingly, the vibrational mode around

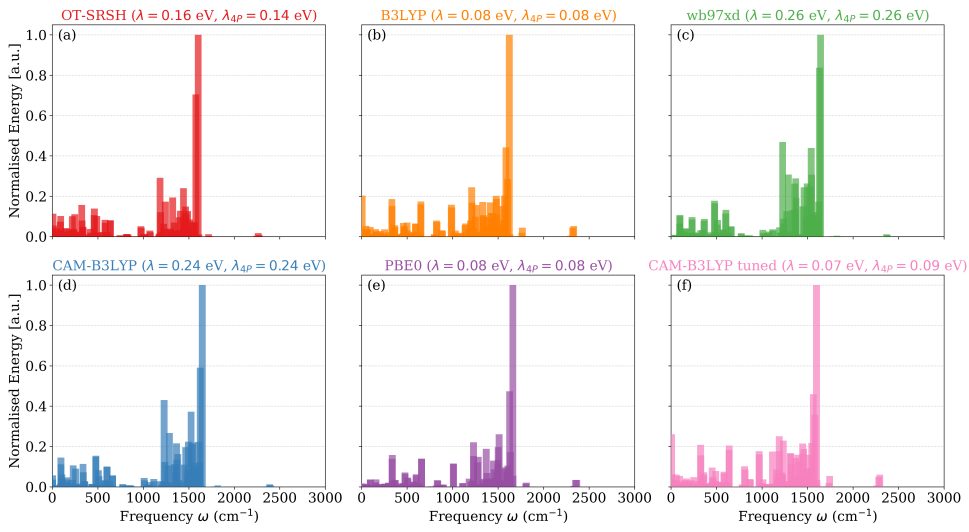

FIG. S11. Normal mode contribution to the overall reorganisation energy across different functionals. For each functional the values are normalised to the mode that couples the strongest.

1350 cm^{-1}, primarily attributed to stretching motions within the acceptor regions, exhibits a significant relative contribution for the ωB97XD and CAM-B3LYP functionals, registering as the second largest component. In contrast, this mode's contribution is notably smaller when calculated with the other functionals.

S5. DIMER AGGREGATE PROPERTIES

In Figs. S12 and S13 we show the behaviour of the different contact pair dimers in the Y6 crystal. In work by Giannini *et al.* [S4] dimers D1 and D4 are considered H aggregates, and D2 is a J aggregate. This classification is based on Kasha's model, where only the relative orientation of the monomer dipole moments is considered. The D4 dimer is distinct from the other contact dimers, as it has little spatial overlap of the monomers. In previous work [S19] this type of dimer has been classified as a V aggregate. In the case of the contact dimers of Y6 our analysis below shows this difference in classification is warranted.

For all six different types of dimers considered in Figs. S12 and S13, we observe that the four lowest energy triplet states are all FE states ($\omega_{CT} < 0.25$) followed by 2 higher in energy CT states ($\omega_{CT} > 0.75$), with a small amount of CT-FE mixing which slightly depends on the dimer considered. The extent to which the triplet states are delocalised across the dimer (ω_{PR}) depends strongly on the dimer considered. The triplet states in e.g. dimer D2 are entirely delocalised across both monomer units with $\omega_{PR} \simeq 2$, while the dimer D4 triplet states are nearly completely localised on a single monomer unit, $\omega_{PR} \simeq 1$.

In contrast, Figs. S12 and S13 show that for dimers D1, D2, D3, D5 and D6 the two lowest energy singlet states are CT states, followed by two higher in energy FE states. For the dimer D4 all four lowest energy states are essentially mixed CT-FE states. The OT-SRSH functional also predicts that the degree of CT-FE mixing is greater for all singlet states than for the triplet states. For all the dimers considered the four lowest energy states are significantly delocalised across both monomer units, with the singlet states on dimer D2 being completely delocalised with $\omega_{PR} \simeq 2$, while the states on dimer D4 are more localised with $\omega_{PR} \simeq 1.6$. Additionally, the singlet states for some dimers are more delocalised than their triplet counterparts.

We consider the more significant difference between the singlet and triplet states to be the extent of CT-FE mixing between states. This clearly plays a role in the larger shift in energy of the singlet than triplet states. This goes beyond the view that this difference is solely due to the difference in the singlet and triplet extent of delocalisation. To further illustrate this, we consider the D2 dimer: both the singlet and triplet states are fully delocalised across the dimer. The cause of the larger gap in energy of the two lowest energy FE singlet states than the lowest energy FE triplet states can therefore only be due to the difference in CT-FE mixing.

To explain some of the different behaviour of the dimers, we considered the correlation between the electron and hole of a given exciton on fragments of the dimer. The structures of the different dimers (shown in figs. S17, S18 and S19) show the H (co-facial), J (head-tail) and V (oblique) orientations. There is significant wavefunction overlap in dimers D1 and D2, but limited overlap for dimer D4.

Fig. S12 shows that when there is overlap between the two core (donor) units, that the S_1 state is a CT state, whilst the S_3 state is an FE state. However, when there is limited overlap between the two donor units, as in dimer D4, Fig. S12 shows that the 4 lowest energy singlet states are mixed FE-CT states.

To understand this, we first consider the monomer S_1 state. Shown in Fig. S14, the predominant contributions of the S1 state have FE character, with the electron and hole both localised on core (donor) unit. There are minor CT contributions from when either the electron or hole are localised on the core or end units respectively.

In dimer D2, where there is overlap between the two core units, Fig. S16 shows that S_3, an FE state, is a delocalised state. The main FE contributions are observed on the core of each monomer. The contributions with CT character are minor, and occur with the electron and hole on the same monomer. By contrast, the main contribution for the CT state, S_1, on dimer D2 arises from when the electron and hole are on core units of different monomers. We, therefore, see that when there is overlap between the D units, the electronic structure of the FE and CT states are essentially a delocalised version of the electronic structure of the monomer, and a charge-separated version of the electronic structure of the monomer, facilitated by significant overlap. Here, the unique properties of Y6 mean the charge-transfer state is lower in energy than the FE state.

However, in dimer D4, where there is limited overlap between the two core units Fig. S12 shows that all 4 lowest energy singlet states are mixed FE-CT states. In dimer D4, contact between the monomers is exclusively between two end (acceptor) units ($A'1$ and $A'2$). For

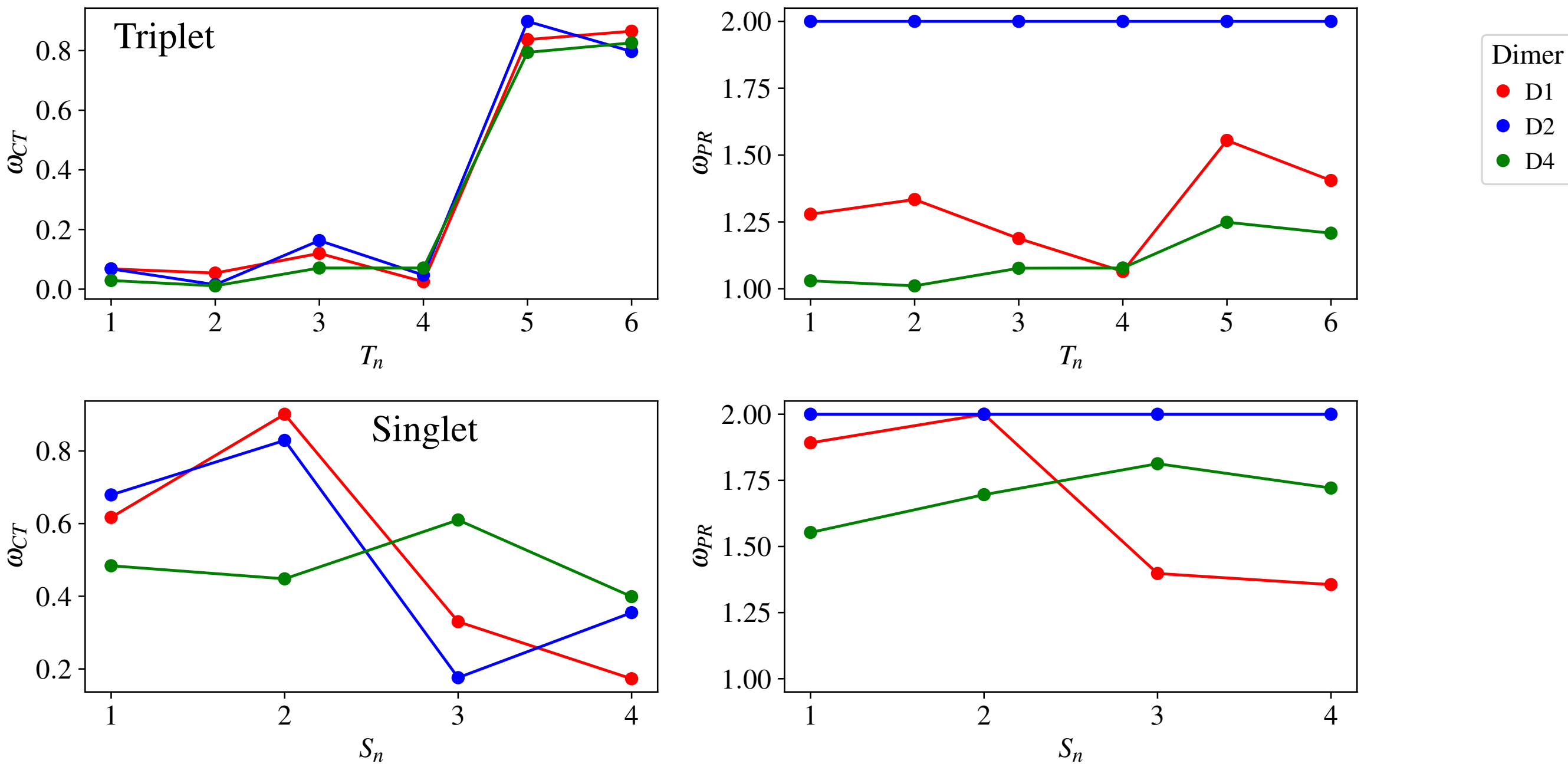

FIG. S12. The extent of charge transfer (ω_{CT}) and extent of delocalisation (ω_{PR}) of the singlet and triplet states of the D1, D2 and D4 dimer.

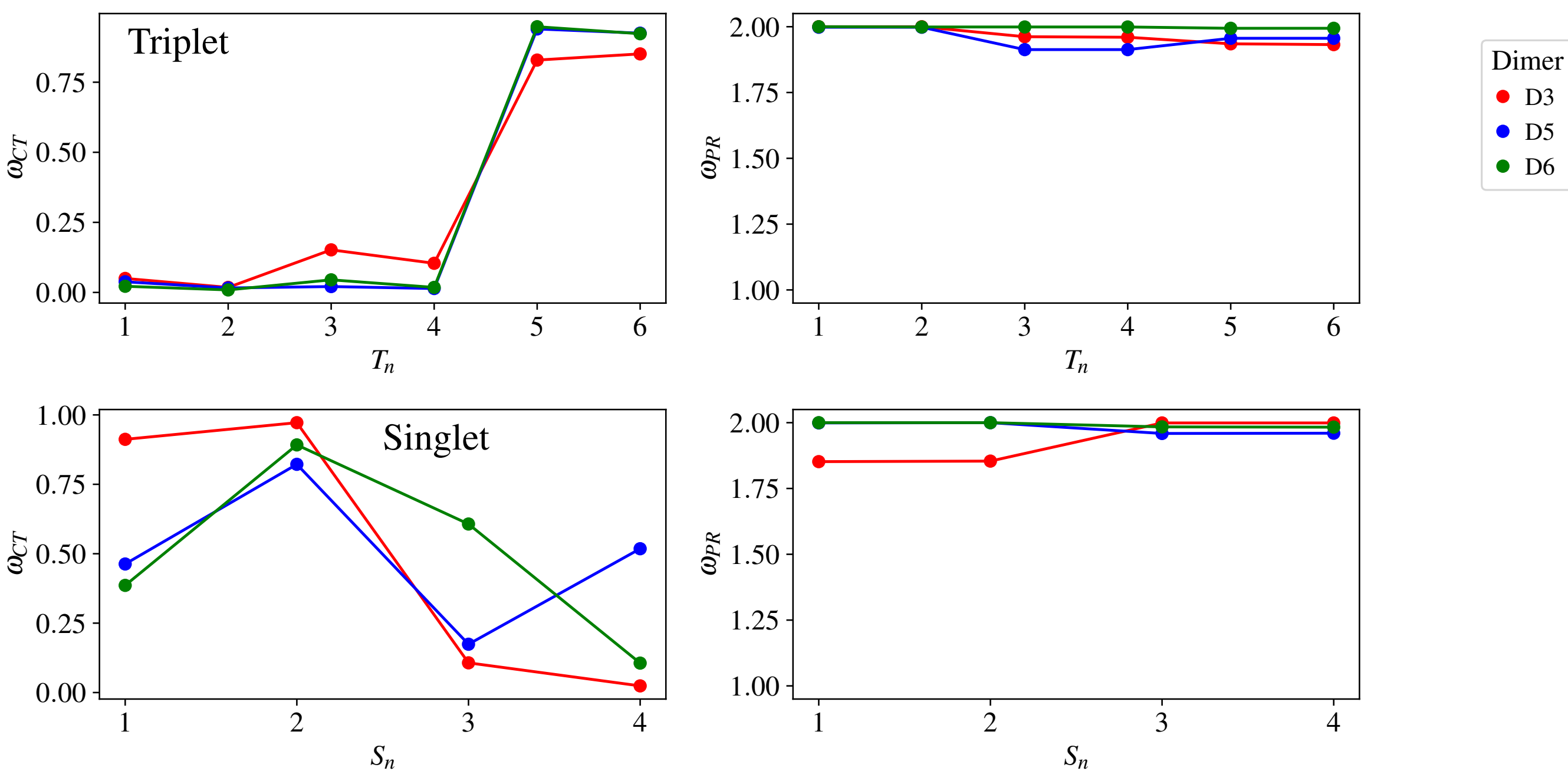

FIG. S13. The extent of charge transfer (ω_{CT}) and extent of delocalisation (ω_{PR}) of the singlet and triplet states of the D3 (H aggregate), D5 (J aggregate) and D6 (J aggregate).

parts of the monomer wavefunction when both electron and hole are on donor unit $D2$ or when either the electron or hole are on $D2$ and the other is on the acceptor $A2$ there is limited delocalisation of the wavefunction in the dimer, as these regions of the wavefunctions in dimer D2 do not overlap significantly with the wavefunction of dimer D1.

By contrast, when either the electron or hole are on $D2$ and the other is on the acceptor $A'2$, there is overlap of wavefunction of dimer D1 facilitating the formation of a CT like state. This means that for D4 the S1 state is a mixture of localised FE state on D2 and a CT state

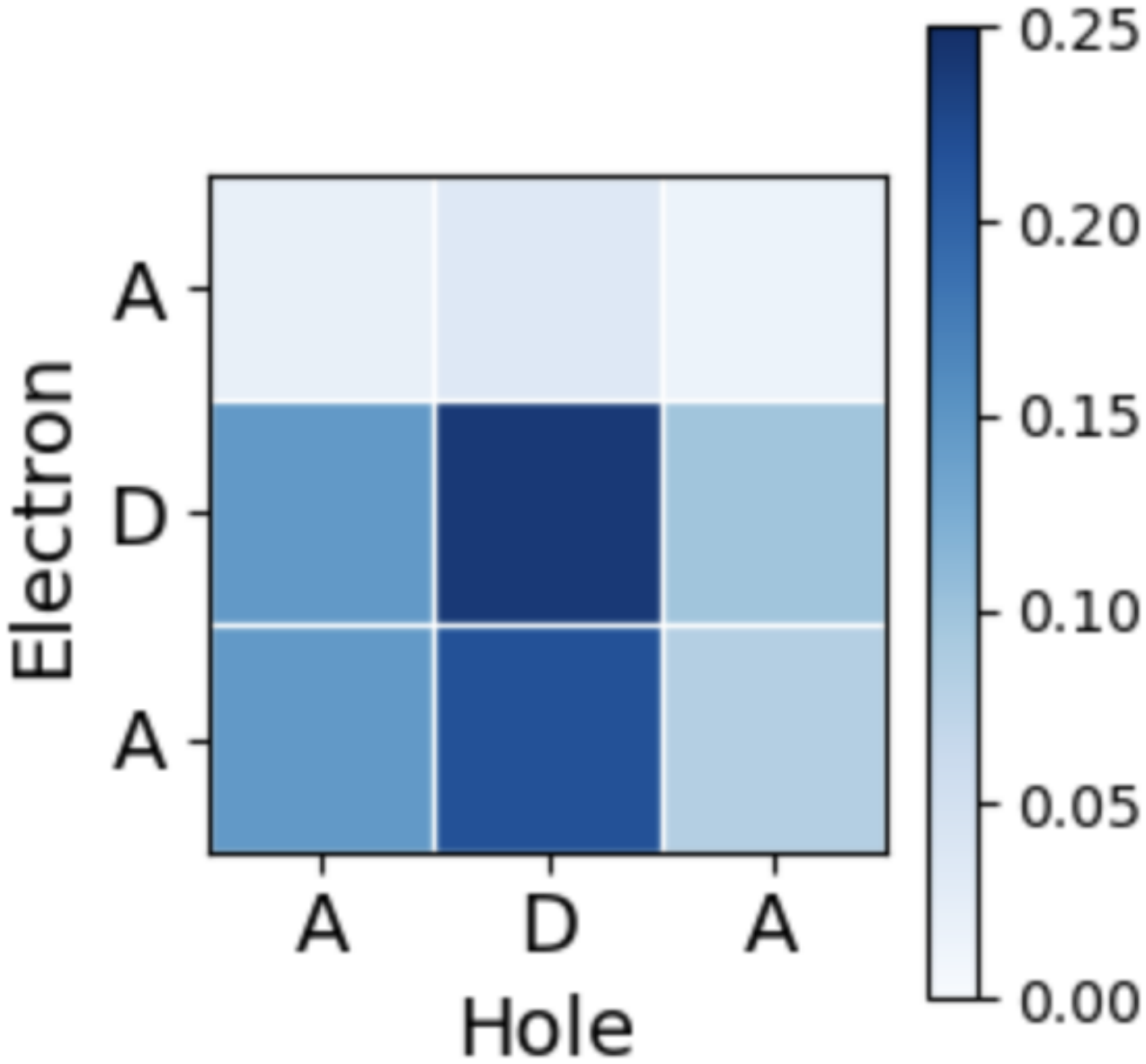

FIG. S14. Electron/Hole correlation plot of the S_1 state for the Y6 monomer. The monomer is fragmented into two end acceptor units and a central donor unit. The fragmentation scheme is shown in Fig. (S15). The colourbar corresponds to the 1 particle transition density (given in terms of the electron and hole position) for the given excited state. On-diagonal elements correspond to the electron and hole being correlated on the same fragment, e.g. a Frenkel Exciton and off-diagonal elements correspond to Charge-Transfer excitation.

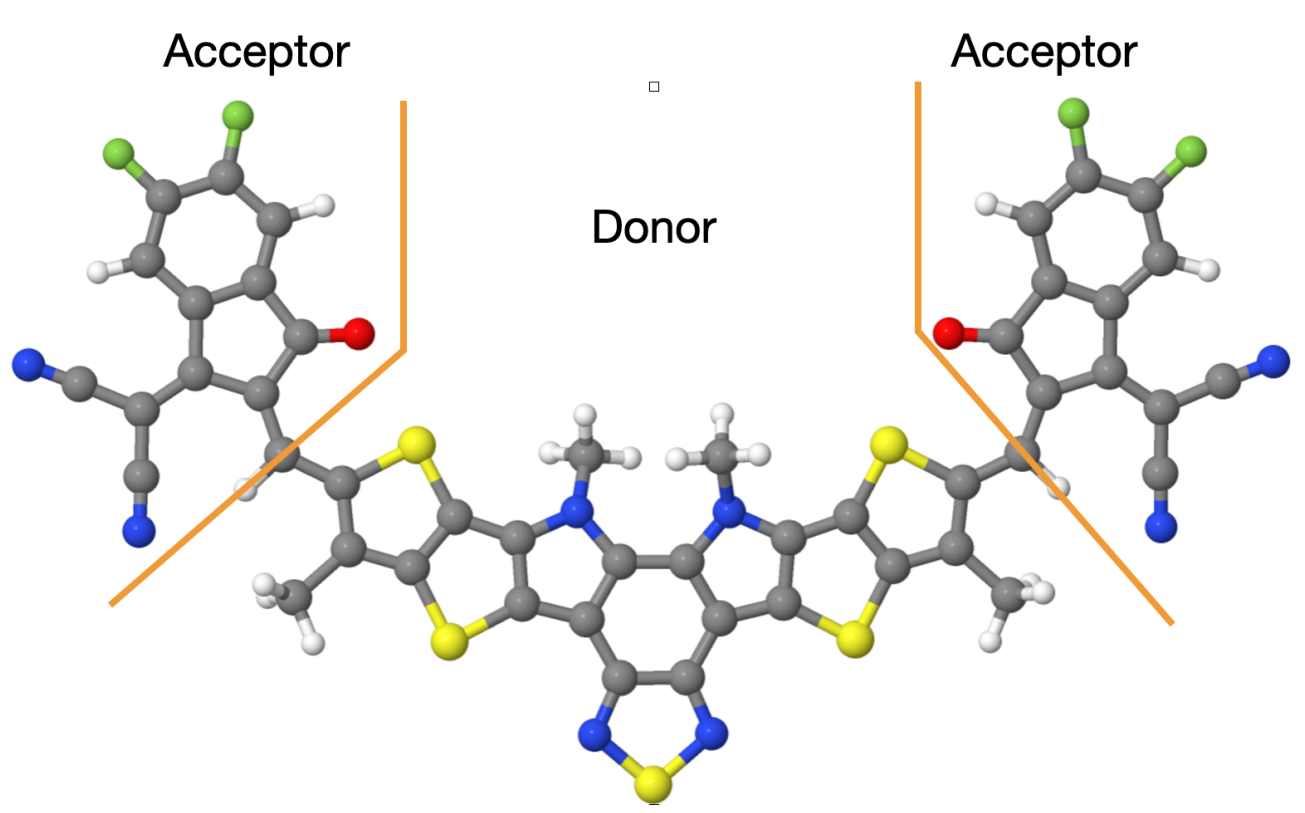

FIG. S15. Fragmentation scheme of the Y6 monomer.

from electron or hole transfer across from the $A'1$ to $A'2$ fragments. Since the S1 and S2 state are mixed FE-CT states, the S3 and S4 states also have mixed character.

S6. OTHER DIMERS

We here show the three different types of dimer found in the Y6 crystal structure namely, the H aggregate D1 dimer (Fig S17), the J aggregate D2 dimer (Fig S18) and the V aggregate D4 dimer (Fig S19).

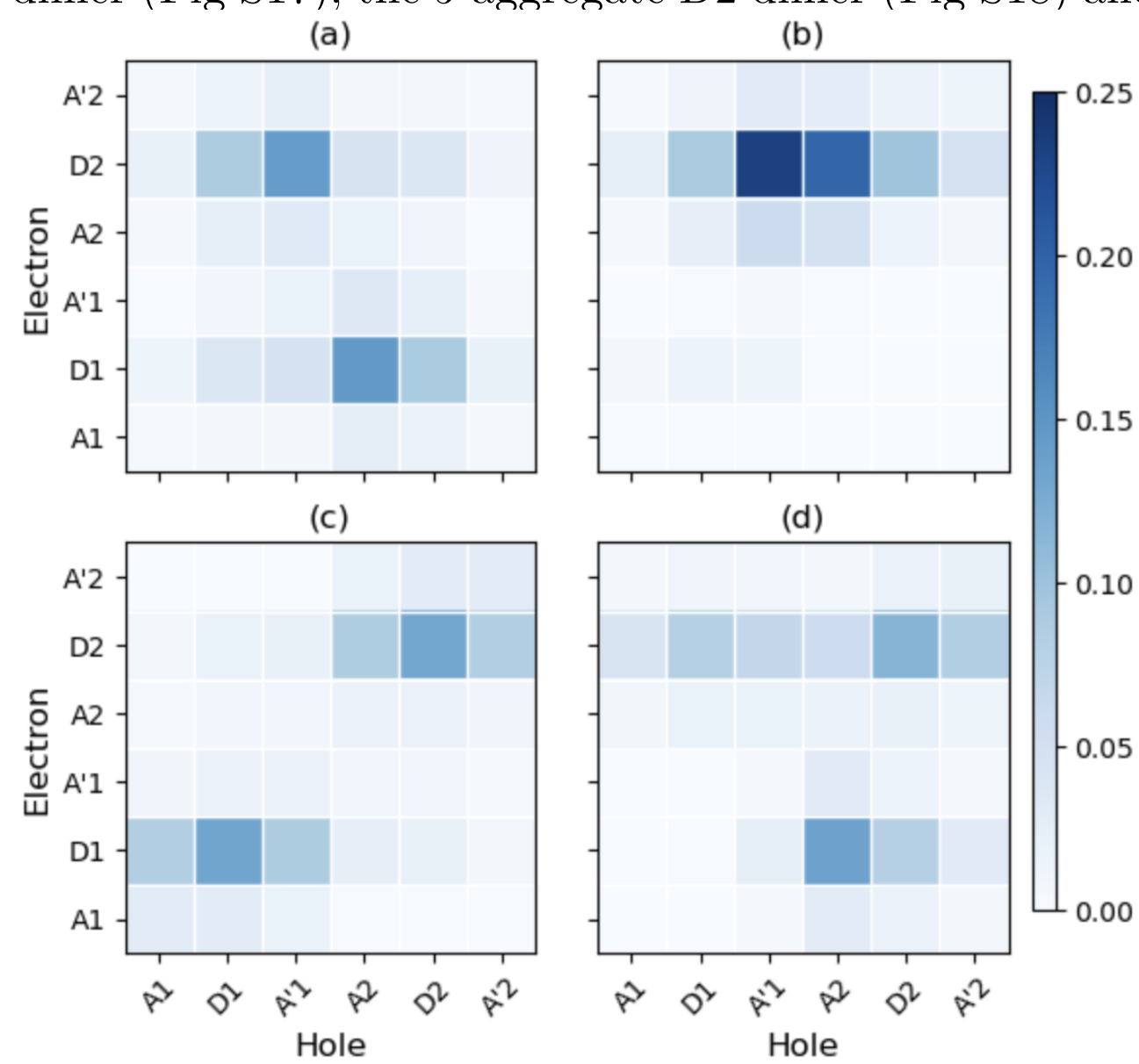

FIG. S16. Electron/Hole correlation plots of the S_1 (top left) and S_3 (bottom left) states on dimer D2, and the S_1 (top right) and S_3 (bottom right) states dimer D4 . Monomers within each dimer are fragmented into two (acceptor) end units and a core (donor) unit, denoted as A/A' and D respectively. Results obtained from electronic structure calculations using OT-LC-ωhPBE/6-31G(d,p) and wavefunction analysis carried out using the Theodore3.2 package [S20]. The colourbar again corresponds to the 1 particle transition density (given in terms of the electron and hole position) for the given excited state. On-diagonal elements correspond to the electron and hole being correlated on the same fragment, e.g. a Frenkel Exciton and off-diagonal elements correspond to Charge-Transfer excitation.

[S1] P. Fürk, S. Mallick, T. Rath, M. Reinfelds, M. Wu, E. Spiecker, N. Simic, G. Haberfehlner, G. Kothleitner, B. Ressel, *et al.*, The challenge with high permittivity acceptors in organic solar cells: a case study with y-series derivatives, Journal of Materials Chemistry C **11**, 8393 (2023).

[S2] P. Li, J. Fang, Y. Wang, S. Manzhos, L. Cai, Z. Song, Y. Li, T. Song, X. Wang, X. Guo, *et al.*, Synergistic effect of dielectric property and energy transfer on charge separation in non-fullerene-based solar cells, Angewandte Chemie International Edition **60**, 15054 (2021).

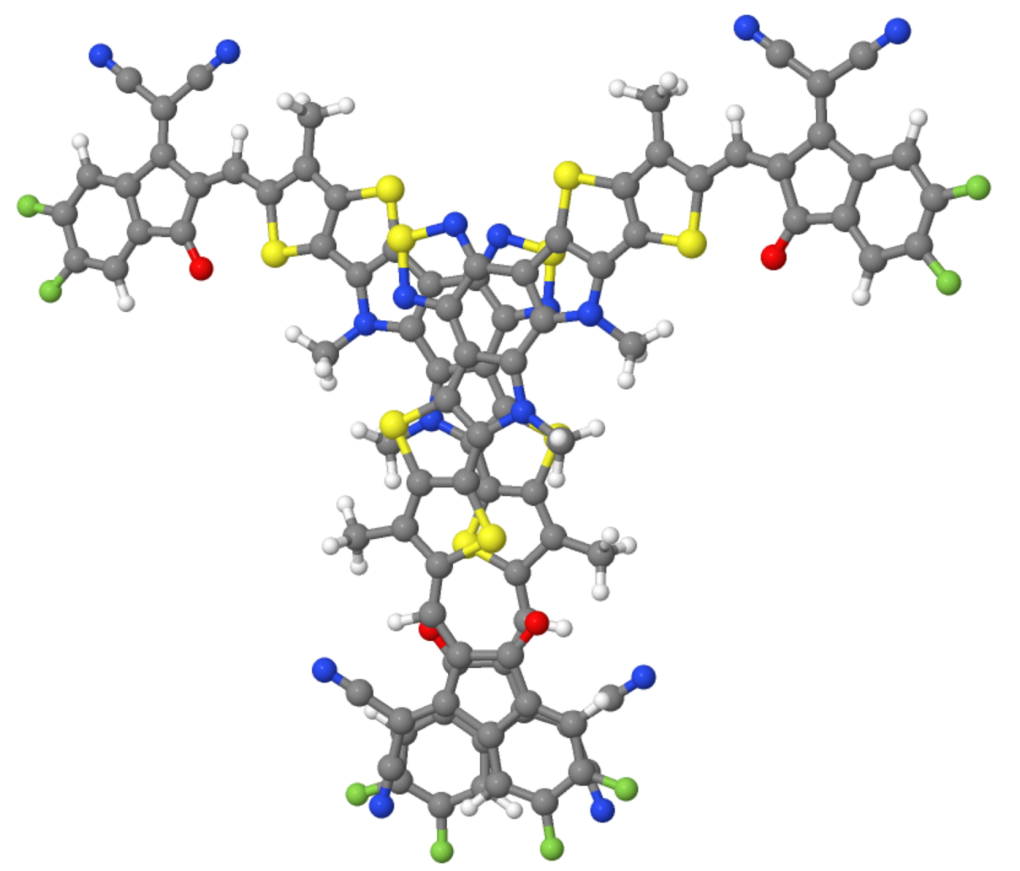

FIG. S17. D1 dimer as an example of a H aggregate in Y6.

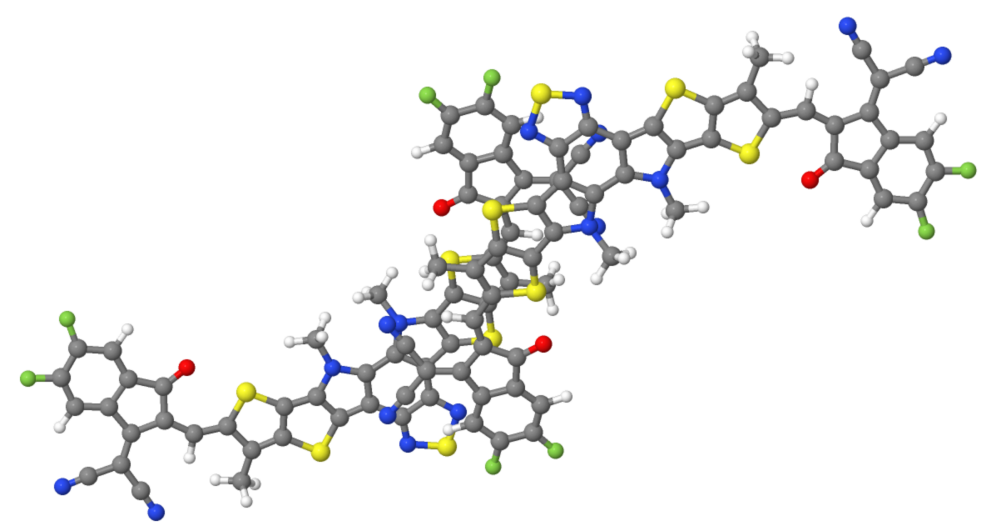

FIG. S18. D2 dimer as an example of a J aggregate in Y6.

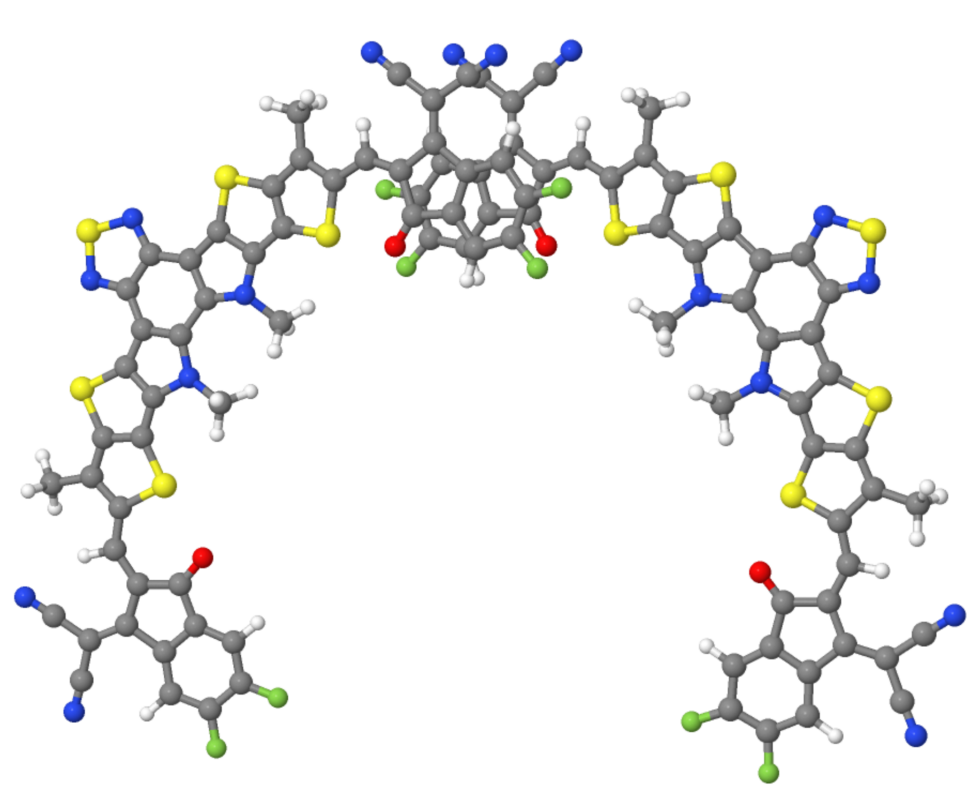

FIG. S19. D4 dimer as an example of a V aggregate in Y6.

[S3] R. Kerremans, C. Kaiser, W. Li, N. Zarrabi, P. Meredith, and A. Armin, The optical constants of solution-processed semiconductors—new challenges with perovskites and non-fullerene acceptors, Advanced Optical Materials **8**, 2000319 (2020).

[S4] S. Giannini, D. J. Sowood, J. Cerdá, S. Frederix, J. Grüne, G. Londi, T. Marsh, P. Ghosh, I. Duchemin, N. C. Greenham, *et al.*, On the role of charge transfer excitations in non-fullerene acceptors for organic photovoltaics, Materials Today **80**, 308 (2024).

[S5] S. J. Akram, S. Meißner, and S. Kümmel, Analyzing electronic excitations and exciton binding energies in y6 films, Advanced Functional Materials , 2419236 (2025).

[S6] C. Halsey-Moore, P. Jena, and J. T. McLeskey, Tuning range-separated dft functionals for modeling the peak absorption of meh-ppv polymer in various solvents, Computational and Theoretical Chemistry **1162**, 112506 (2019).

[S7] N. Bogo and C. J. Stein, Benchmarking dft-based excited-state methods for intermolecular charge-transfer excitations, Physical Chemistry Chemical Physics **26**, 21575 (2024).

[S8] S. H. Jeon, S. Kang, and T. Kim, Exploring an adequate dft functional to accurately describe the singlet excited state properties in hlct materials: A benchmark study, Advanced Theory and Simulations **7**, 2300363 (2024).

[S9] J.-L. Lan, X.-N. Liu, C.-N. Xiao, M.-Y. Sui, and G.-Y. Sun, Correction of the calculation method of ct state energy in itic and y6 acceptor systems, Journal of Photochemistry and Photobiology A: Chemistry **456**, 115821 (2024).

[S10] R. Magyar and S. Tretiak, Dependence of spurious charge-transfer excited states on orbital exchange in tddft: large molecules and clusters, Journal of chemical theory and computation **3**, 976 (2007).

[S11] A. Olaya-Castro and G. D. Scholes, Energy transfer from förster–dexter theory to quantum coherent light-harvesting, International Reviews in Physical Chemistry **30**, 49 (2011).

[S12] M. Azzouzi, J. Yan, T. Kirchartz, K. Liu, J. Wang, H. Wu, and J. Nelson, Nonradiative energy losses in bulk-heterojunction organic photovoltaics, Physical Review X **8**, 10.1103/physrevx.8.031055 (2018).

[S13] C. Wang, H. Dong, W. Hu, Y. Liu, and D. Zhu, Semiconducting -conjugated systems in field-effect transistors: A material odyssey of organic electronics, Chemical Reviews **112**, 2208–2267 (2011).

[S14] K.-H. Lin, A. Prlj, L. Yao, N. Drigo, H.-H. Cho, M. K. Nazeeruddin, K. Sivula, and C. Corminboeuf, Multiarm and substituent effects on charge transport of organic hole transport materials, Chemistry of Materials **31**, 6605–6614 (2019).

[S15] S. F. Nelsen, S. C. Blackstock, and Y. Kim, Estimation of inner shell marcus terms for amino nitrogen compounds by molecular orbital calculations, Journal of the American Chemical Society **109**, 677–682 (1987).

[S16] J. R. Reimers, A practical method for the use of curvilinear coordinates in calculations of normal-mode-projected displacements and duschinsky rotation matrices for large molecules, The Journal of Chemical Physics **115**, 9103–9109 (2001).

[S17] H. Yang, M. Azzouzi, L. Lindh, J. S. Muller, T. C. Rein, F. D. Eisner, J. M. Frost, and J. Nelson, Unveiling fused-ring electron acceptors' excited-state properties via bayesian inference and temperature-dependent spectroscopy, in preparation (2026).

[S18] S. Kashani, Z. Wang, C. Risko, and H. Ade, Relating reorganization energies, exciton diffusion length and non-radiative recombination to the room temperature uv-vis absorption spectra of nf-sma, Materials Horizons **10**, 443–453 (2023).

[S19] G. Han, T. Hu, and Y. Yi, Reducing the singlet-triplet energy gap by end-group stacking toward high-efficiency organic photovoltaics, Advanced Materials **32**, 10.1002/adma.202000975 (2020).

[S20] F. Plasser, Theodore: A toolbox for a detailed and automated analysis of electronic excited state computations, The Journal of chemical physics **152** (2020).
